# Mutations in the splicing regulator Prp31 lead to retinal degeneration in *Drosophila*

**DOI:** 10.1242/bio.052332

**Published:** 2021-01-25

**Authors:** Sarita Hebbar, Malte Lehmann, Sarah Behrens, Catrin Hälsig, Weihua Leng, Michaela Yuan, Sylke Winkler, Elisabeth Knust

**Affiliations:** Max-Planck-Institute of Molecular Cell Biology and Genetics, Pfotenhauerstrasse 108, 01307 Dresden, Germany

**Keywords:** Spliceosome, Photoreceptor cells, Rhodopsin, *scarlet*, *twinfilin*

## Abstract

Retinitis pigmentosa (RP) is a clinically heterogeneous disease affecting 1.6 million people worldwide. The second-largest group of genes causing autosomal dominant RP in human encodes regulators of the splicing machinery. Yet, how defects in splicing factor genes are linked to the aetiology of the disease remains largely elusive. To explore possible mechanisms underlying retinal degeneration caused by mutations in regulators of the splicing machinery, we induced mutations in *Drosophila Prp31,* the orthologue of human *PRPF31*, mutations in which are associated with RP11. Flies heterozygous mutant for *Prp31* are viable and develop normal eyes and retina. However, photoreceptors degenerate under light stress, thus resembling the human disease phenotype. Degeneration is associated with increased accumulation of the visual pigment rhodopsin 1 and increased mRNA levels of *twinfilin*, a gene associated with rhodopsin trafficking. Reducing rhodopsin levels by raising animals in a carotenoid-free medium not only attenuates rhodopsin accumulation, but also retinal degeneration. Given a similar importance of proper rhodopsin trafficking for photoreceptor homeostasis in human, results obtained in flies presented here will also contribute to further unravel molecular mechanisms underlying the human disease.

This paper has an associated First Person interview with the co-first authors of the article.

## Abstract (precis)

**Summary:** Retinitis pigmentosa (RP) is a human disease resulting in blindness, which affects 1 in 4.000 people worldwide. So far >90 genes have been identified that are causally related to RP. Mutations in the splicing factor PRPF31 are linked to RP11. We induced mutations in the *Drosophila* orthologue *Prp31* and show that flies heterozygous for *Prp31* undergo light-dependent retinal degeneration. Degeneration is associated with increased accumulation of the light-sensitive molecule, rhodopsin 1. In fact, reducing rhodopsin levels by dietary intervention modifies the extent of retinal degeneration. This model will further contribute to better understand the aetiology of the human disease.

## INTRODUCTION

Retinitis pigmentosa (RP; OMIM 268000) is a clinically heterogeneous group of retinal dystrophies, which affects more than one million people worldwide. It often starts with night blindness in early childhood, continues with the loss of the peripheral visual field (tunnel vision), and progresses to complete blindness in later life due to gradual degeneration of photoreceptor cells (PRCs). RP is a genetically heterogeneous disease and can be inherited as autosomal dominant (adRP), autosomal recessive (arRP) or X-linked (xlRP) disease. So far >90 genes have been identified that are causally related to non-syndromic RP (Ali et al., 2017; Verbakel et al., 2018). Affected genes are functionally diverse. Some of them are expressed specifically in PRCs and encode, among others, transcription factors (e. g. *CRX*, an *otx*-like photoreceptor homeobox gene), components of the light-induced signalling cascade, including the visual pigment rhodopsin (Rho/*RHO* in *Drosophila*/human), or genes controlling vitamin A metabolism (e.g. *RLBP-1*, encoding Retinaldehyde-binding protein). Other genes are associated with a more general control of cellular homeostasis, for example genes involved in trafficking or cell polarity (e.g. *CRB1*) [reviewed in (Daiger et al., 2014, 2013; Hollingsworth and Gross, 2012; Nemet et al., 2015)]. Interestingly, the second-largest group of genes causing adRP, comprising 7 of 25 genes known, encodes regulators of the splicing machinery. So far, mutations in five pre-mRNA processing factor (PRPF) genes, PRPF3, PRPF4, PRPF6, PRPF8 and PRPF31, have been linked to adRP, namely RP18, RP70, RP60, RP13 and RP11, respectively. Pim1-associated protein (PAP1) and small nuclear ribonuclearprotein-200 (SNRNP200), two genes also involved in splicing, have been suggested to be associated with RP9 and RP33, respectively (Maita et al., 2004; Zhao et al., 2009) [reviewed in (Liu and Zack, 2013; Mordes et al., 2006; Poulos et al., 2011; Ruzickova and Stanek, 2016)]. The five *PRPF* genes encode components regulating the assembly of the U4/U6.U5 tri-snRNP, a major module of the pre-mRNA spliceosome machinery (Nguyen et al., 2015; Patel and Bellini, 2008; Will and Luhrmann, 2011). Several hypotheses have been put forward to explain why mutations in ubiquitously expressed components of the general splicing machinery show a dominant phenotype only in the retina. One hypothesis suggests that PRCs with only half the copy number of a gene encoding a general splicing component cannot cope with the elevated demand of RNA-/protein synthesis required to maintain the exceptionally high metabolic rate of PRCs in comparison to other tissues. Hence, halving their gene dose eventually results in apoptosis. Although this model is currently favoured, other mechanisms, such as impaired splicing of PRC-specific mRNAs or toxic effects caused by accumulation of mutant proteins have been discussed and may contribute to the disease phenotype [discussed in (Mordes et al., 2006; Scotti and Swanson, 2016; Tanackovic et al., 2011)]. More recent data obtained from retinal organoids established from RP11 patients showed that removing one copy of *PRPF31* affects the splicing machinery specifically in retinal and retinal pigment epithelial (RPE) cells, but not in patient-derived fibroblasts or iPS cells (Buskin et al., 2018).

The observation that all adRP-associated genes involved in splicing are highly conserved from yeast to human allows to use model organisms to unravel the genetic and cell biological functions of these genes in order to obtain mechanistic insight into the origin of the diseases. In the case of RP11, the disease caused by mutations in *PRPF31*, three mouse models have been generated by knock-in and knock-out approaches. Unexpectedly, mice heterozygous mutant for a null allele or a point mutation that recapitulates a mutation in the corresponding human gene did not show any sign of retinal degeneration in 12- and 18-month-old mice, respectively (Bujakowska et al., 2009). Further analyses revealed that the retinal pigment epithelium, rather than the PRCs, is the primary tissue affected in *Prpf31* heterozygous mice (Farkas et al., 2014; Graziotto et al., 2011; Hamieh and Nandrot, 2019). Other data show that homozygous *PRPF31* mice are not viable (Dickinson et al., 2016). Morpholino-induced knockdown of zebrafish *Prpf31* results in strong defects in PRC morphogenesis and survival (Linder et al., 2011). Defects induced by retina-specific expression of zebrafish *Prpf31* constructs that encode proteins with the same mutations as those mapped in RP11 patients (called AD5 and SP117) were explained to occur by either haplo-insufficiency or by a dominant-negative effect of the mutant protein (Yin et al., 2011). In *Drosophila*, no mutations in the orthologue *Prp31* have been identified so far. RNAi-mediated knockdown of *Prp31* in the *Drosophila* eye results in abnormal eye development, ranging from smaller eyes to complete absence of the eye, including loss of PRCs and pigment cells (Ray et al., 2010).

In order to get better insights into the mechanisms by which *Prp31* prevents retinal degeneration we aimed to establish a meaningful *Drosophila* model for RP11-associated retinal degeneration. Therefore, we isolated two mutant alleles of *Prp31*, *Prp31^P17^* and *Prp31^P18^*, which carry missense mutations affecting conserved amino acids. Flies heterozygous for either of these mutations are viable and develop normally. Strikingly, when exposed to constant light, these mutant flies undergo retinal degeneration, thus mimicking the disease phenotype of RP11 patients. Degeneration of mutant PRCs is associated with accumulation and abnormal distribution of the visual pigment rhodopsin, Rh1, in PRCs. Reduction of dietary vitamin A, a precursor of the chromophore 11-cis-3-hydroxyretinal, which binds to opsin to generate the functional rhodopsin, mitigates both aspects of the mutant phenotype, rhodopsin accumulation and retinal degeneration. From this we conclude that Rh1 accumulation and/or misdistribution reflect a degeneration-prone condition in the *Prp31* mutant retina.

## RESULTS

### Two *Prp31* alleles were discovered by TILLING

It was recently shown that RNAi-mediated knockdown of *Drosophila Prp31* in the eye using eye-specific Gal4-lines [*eyeless* (*ey*)-Gal4 or GMR-Gal4] results in abnormal eye development, ranging from smaller eyes to complete absence of the eye, including loss of photoreceptor cells (PRCs) and pigment cells (Ray et al., 2010). Both Gal4-lines are expressed throughout eye development. Therefore, some of the defects observed could be the result of impaired early development of the eye, such as defective cell fate specification, which would only indirectly affect PRC development. Here, we aimed to establish a more meaningful *Drosophila* model for RP11-associated retinal degeneration, a human disease associated with mutations in the human orthologue *PRPF31*, which would allow a deeper insight into the role of this splicing factor in the origin and progression of the disease.

**Fig. 1.**
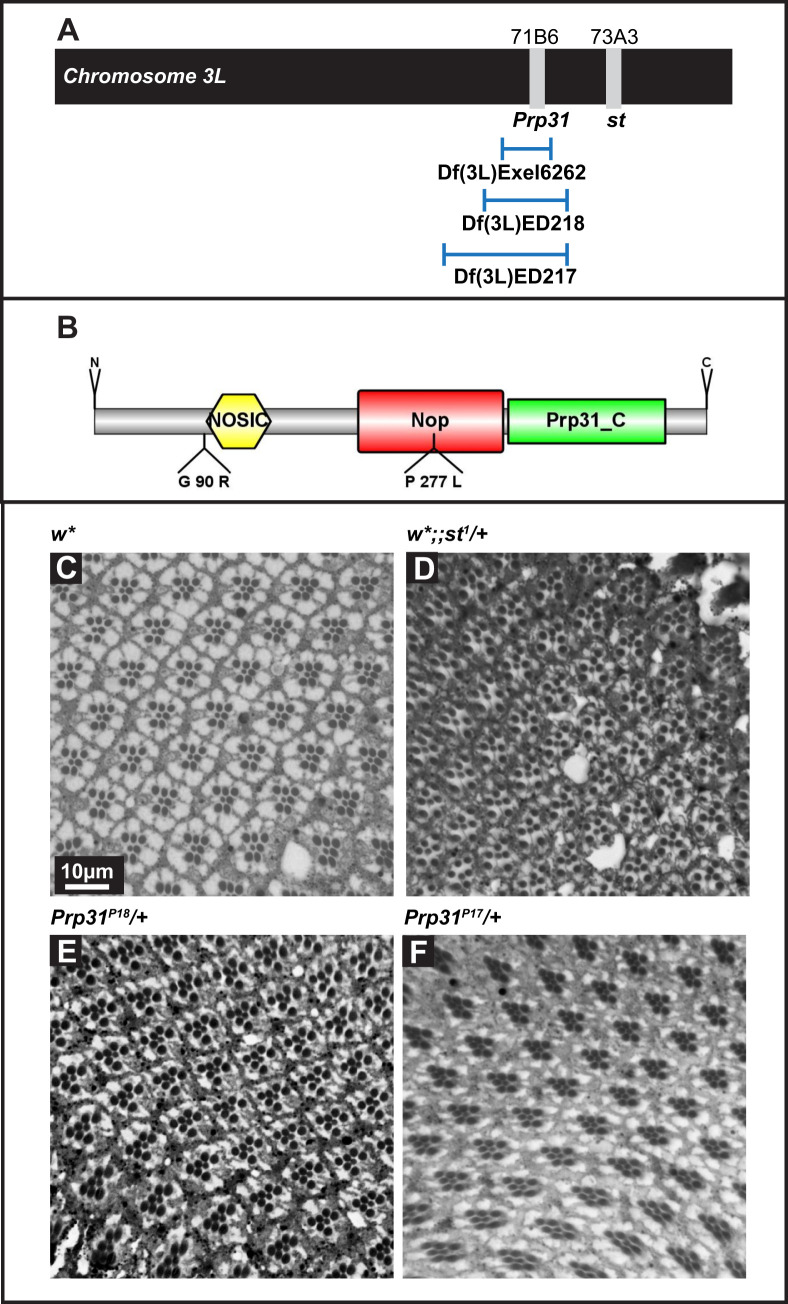
**Eyes of *Prp31* mutant flies have no gross morphological abnormalities at eclosion.** (A) Schematic of chromosome arm 3L. *Prp31* and *scarlet* (*st*) are situated 2 cM apart (3–42 and 3–44, respectively; cytological positions 71B6 and 73A3, respectively; www.flybase.org). In both *Prp31* mutant alleles the marker *st*^1^ from the original mutagenised chromosome (*ru st e ca*) is retained. The three deficiencies used cover the *Prp31* locus, but not the *st* locus. (B) Schematic overview of the *Drosophila* Prp31 protein. The figure is drawn to scale using IBS (Liu et al., 2015). Domains described here are indicated. The two *Prp31* alleles studied here carry non-conservative missense mutations, G90R in *Prp31^P17^* and P277L in *Prp31^P18^*. (C–F) Representative bright-field images of Toluidine-blue stained semi-thin sections of eyes of w* (C), w*;; st^1^/+ (D) *Prp31^P18^ /+* (E) and *Prp31^P17^ /+* (F). Complete genotypes can be found in Table S1. Upon eclosion, flies were kept for 2 days under regular light conditions. Note that the number and stereotypic arrangement of photoreceptor cells within the mutant ommatidia are not affected. Scale bar, 10 µm.

Therefore, we set out to isolate specific mutations in *Drosophila Prp31* by targeting induced local lesions in genomes (TILLING), following a protocol described recently (Spannl et al., 2017)*.* In total, 2.400 genomes of ethyl methanesulfonate (EMS)-mutagenised flies were screened for sequence variants in two different amplicons of *Prp31*. Four sequence variants were identified, which were predicted to result in potentially deleterious missense mutations. Two of the four lines, named *Prp31^P17^* and *Prp31^P18^*, were recovered from the living fly library and crossed for three generations to control, white-eyed (w*) flies to reduce the number of accompanying sequence variations. We outcrossed the mutants with white-eyes flies (w*) rather than with wild-type, red-eyed flies to generate a sensitised background for light-dependent degeneration experiments, since the presence of the pigment granules surrounding each ommatidium contributes towards lower sensitivity to light (Stark and Carlson, 1984). *Prp31^P18^* flies were viable as homozygotes and in trans over any of three deficiencies, which remove, among others, the *Prp31* locus (Fig. 1A). In contrast, no homozygous *Prp31^P17^* flies were obtained. However, *Prp31^P17^* was viable in trans over *Prp31^P18^* and over *Df(3L)ED217*. This suggests that the lethality was due to a second site mutation, which was not removed during outcrossing. We noticed that outcrossing *Prp31^P17^* and *Prp31^P18^* did not remove *scarlet* (*st*), one of the markers of the original, mutagenised chromosome (*ru st e ca*) mapping close to *Prp31*. Therefore, the correct genotypes of the two mutant lines are w*; *Prp31^P17^, st^1^* and w*; *Prp31^P18^, st^1^*. For simplicity, we will refer to them as *Prp31^P17^* and *Prp31^P18^* throughout the text.

The molecular lesions in the two *Prp31* alleles were mapped in the protein coding region. *Drosophila* PRP31 is a protein of 501 amino acids, which contains a NOSIC domain (named after the central domain of Nop56/SIK1-like protein), a Nucleolar protein (Nop) domain required for RNA binding, a PRP31_C-specific domain and a nuclear localization signal, NLS (Fig. 1B). *Prp31^P17^* contained a point mutation that resulted in a non-conservative glutamine to arginine exchange (G90R) N-terminal to the NOSIC domain. *Prp31^P18^* contained a non-conservative exchange of a proline to a leucine residue in the Nop domain (P277L) (Fig. 1B). Both mutations affect amino acids that are highly conserved in many metazoan species (Fig. S1A). Based on polymorphism phenotyping v2 (PolyPhen-2; http://genetics.bwh.harvard.edu/pph2/) (Adzhubei et al., 2013) analyses, both amino acid substitutions are predicted to be deleterious for the structure and/or function of the protein (Fig. S1B).

### Flies hetero- or hemizygous for *Prp31* undergo light-dependent retinal degeneration

**Fig. 2.**
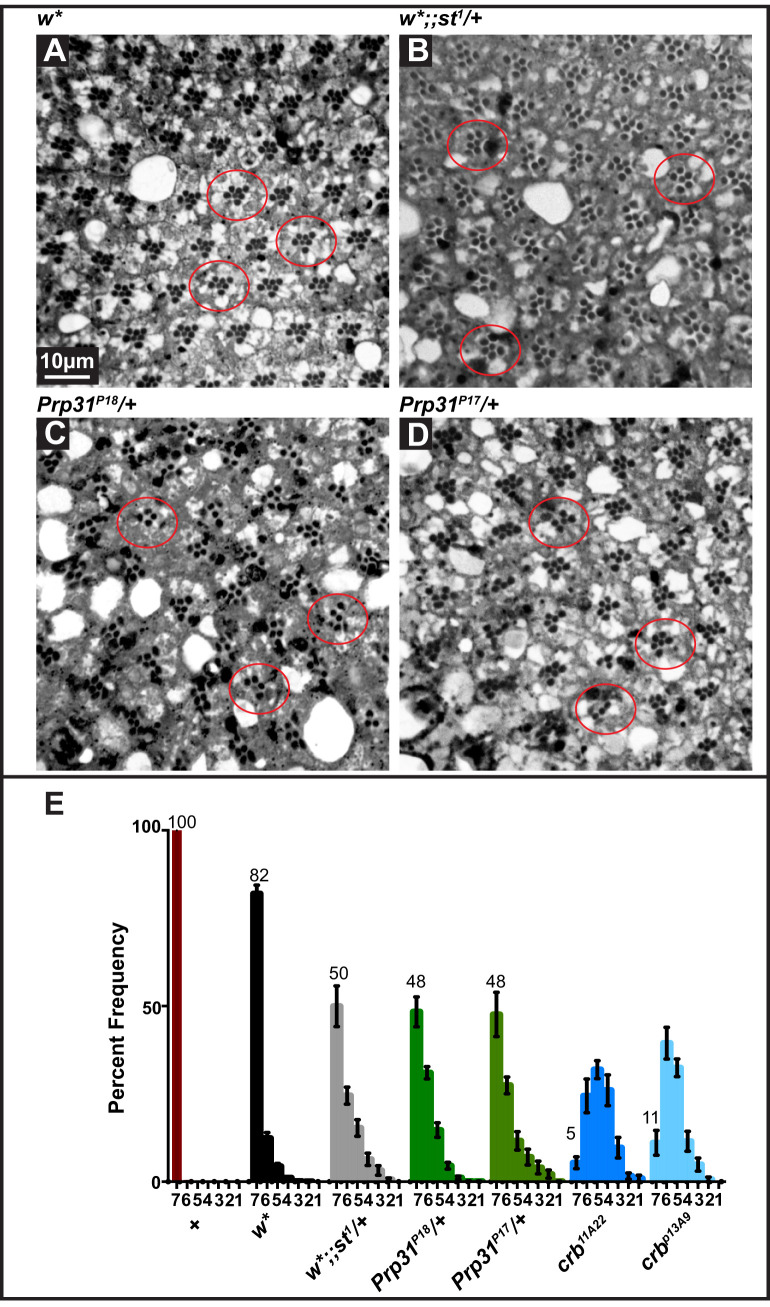
**PRCs of heterozygous *Prp31^P17^* and *Prp31^P18^* flies undergo light-dependent degeneration.** (A–D) Representative bright-field images of Toluidine-blue stained semi-thin sections of eyes of w* (A), w*;; st^1^/+ (B), *Prp31^P18^/+* (C), and *Prp31^P17^ /+* (D). Complete genotypes can be found in Table S1. Upon eclosion, flies were kept for 2 days under regular light conditions and then subjected to a degeneration paradigm of 7 days of continuous, high intensity light exposure. Whereas in w* (A) most ommatidia (red outlines) display seven rhabdomeres indicative of the seven PRCs, w*;; st^1^/+ and *Prp31* mutant ommatidia (B–D, red outlines) display fewer rhabdomeres per ommatidium indicative of degeneration. Scale bar, 10 µm. (E) Quantification of retinal degeneration as indicated by the number of surviving rhabdomeres observed upon high intensity, continuous light exposure. Y-axis: percent frequency of ommatidia displaying one to seven rhabdomeres. Genotypes are indicated below. Number on top of each graph indicates the mean percentage of ommatidia displaying the full complement of seven rhabdomeres. Bars represent mean±s.e.m. (a minimum of *n*=60 ommatidia from eyes of three biological replicates). Statistical significance of differences in this parameter, between genotype pairs, is indicated in Table S2.

Homo- and heterozygous *Prp31^P18^* and heterozygous *Prp31^P17^* animals raised and kept under regular light–dark cycles (12 h light; 12 h dark) have eyes of normal size. Histological sections revealed normal numbers of PRCs per ommatidium (distinguished by the number of rhabdomeres) and a normal stereotypic arrangement of PRCs (Fig. 1C–F and Fig. S2A). This indicates that the development of the retina was not affected by these mutations. However, PRCs of *Prp31^P17^*/+, *Prp31^P18^*/+ and *Prp31^P18^*/*Prp31^P18^* flies showed clear signs of retinal degeneration when exposed to constant light for several days, manifested by a partial or complete loss of rhabdomeric integrity (Fig. 2C,D and Fig. S2B). Quantification of the number of surviving rhabdomeres in *Prp31* mutant retinas revealed only about 48% of ommatidia with the full complement of seven PRCs (Fig. 2E), while w* mutant control flies exhibited 82% of all ommatidia displaying the full complement of rhabdomeres (Fig. 2A,E). The degree of degeneration observed in *Prp31* alleles is less severe and more variable than that observed in the well-established RP12 disease model induced by mutations in the gene *crumbs* (*crb*) (Chartier et al., 2012; Johnson et al., 2002; Spannl et al., 2017). In the two *crb* alleles *crb^11A22^* and *crb^p13A9^* only 5 to 11% of all ommatidia displayed seven rhabdomeres upon exposure to constant light, respectively (Fig. 2E).

**Fig. 3.**
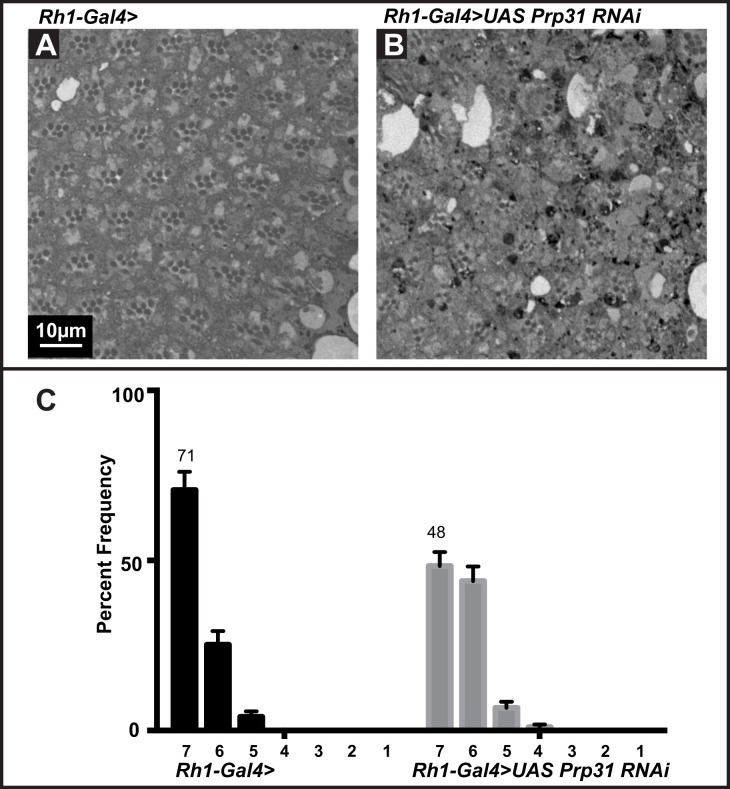
**RNAi-mediated knockdown of *Prp31* results in light-dependent retinal degeneration.** (A,B) Representative bright-field images of Toluidine-blue stained semi-thin sections of eyes of *Rh1-Gal4>* (A; control) and *Rh1-Gal4>UAS Prp31RNAi* (B). Complete genotypes can be found in Table S1. Upon eclosion, flies were kept for 2 days under regular light conditions and then subjected to a degeneration paradigm of 7 days of continuous, high-intensity light exposure. In case of *Rh1-Gal4>UAS Prp31RNAi*, fewer ommatidia with seven rhabdomeres are seen. Scale bar, 10 µm. (C) Quantification of retinal degeneration as indicated by the number of surviving rhabdomeres observed upon high intensity, continuous light exposure. Y-axis: percent frequency of ommatidia displaying one to seven rhabdomeres. Genotypes are indicated below. Number on top of each graph indicates the mean percentage of ommatidia displaying the full complement of seven rhabdomeres. Bars represent mean±s.e.m. (a minimum of *n*=60 ommatidia from eyes of three biological replicates). Whilst 71% of control ommatidia have seven rhabdomeres/ommatidium, this number is significantly reduced to 48% upon knocking-down *Prp31* by RNAi (*P*<0.05, shown in Table S2).

To further confirm that the degeneration phenotype observed in *Prp31^P18^* and *Prp31^P17^* heterozygous flies is due to mutations in *Prp31*, we used additional strategies to reduce/inactivate *Prp31* function*.* First, we knocked down *Prp31* by overexpressing *Prp31* RNAi, mediated by *Rh1*-Gal4, which drives expression late in retinal development, from 70% pupal development into adulthood (Kumar and Ready, 1995). Thereby, we can rule out any early effects on PRC specification or morphogenesis induced by loss of *Prp31*. To make the data comparable to those obtained with *Prp31* alleles (which are in a *w* background), we reduced the red-coloured screening pigments encoded by the *w^+^*-gene on the transgenes by expression of another transgene, GMR-*w^IR^*, which expresses *white* RNAi under the control of the GMR-promoter (Lee and Carthew, 2003). RNAi-mediated knockdown of *Prp31* in PRCs (and concomitant ubiquitous knockdown of *w*) resulted in clear signs of degeneration upon light exposure, such as loss of rhabdomeres and accumulation of intensely stained structures reminiscent to apoptotic bodies (Fig. 3B). In fact, while 71% of control ommatidia revealed 7 identifiable rhabdomeres and no major morphological defects (Fig. 3A,C), the number of ommatidia with a full complement of rhabdomeres decreased to 48% upon induction of *Prp31 RNAi* (Fig. 3C).

**Fig. 4.**
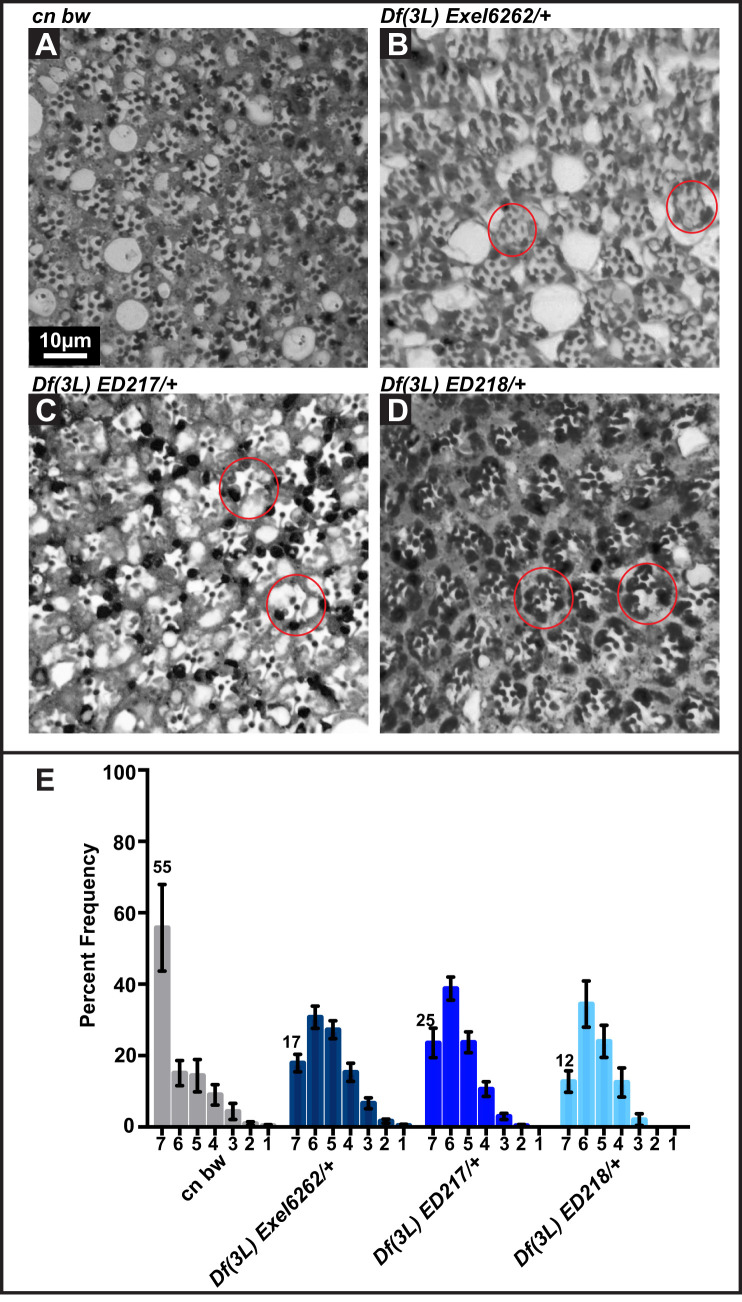
**Flies heterozygous for deficiencies that remove the *Prp31*, but not the *scarlet* locus, undergo light-dependent degeneration.** (A–D) Representative bright-field images of Toluidine-blue stained semi-thin sections of eyes of males of *cn bw* (A), *Df (3L) Exel 6262/+* (B), *Df (3*L*) ED217/+* (C) and *Df (3L) ED218/+* (D). Complete genotypes can be found in Table S1*.* Upon eclosion, flies were kept for 2 days under regular light conditions and then subjected to a degeneration paradigm of 7 days of continuous, high intensity light exposure. Red circles outline individual ommatidia. Scale bar, 10 µm. (E) Quantification of retinal degeneration as indicated by the number of surviving rhabdomeres observed upon high intensity, continuous light exposure. Y-axis: percent frequency of ommatidia displaying one to seven rhabdomeres. Genotypes are indicated below. Number on top of each graph indicates the mean percentage of ommatidia displaying the full complement of seven rhabdomeres. Bars represent mean±s.e.m. (a minimum of *n*=60 ommatidia from eyes of three biological replicates). Statistical significance of differences in this parameter between genotype pairs is indicated in Table S2.

As a second alternative strategy to confirm the role of *Prp31* in retinal degeneration, we analysed the phenotype of three deficiency lines that remove the *Prp31* locus (see Fig. 1A). For a proper comparison with the data obtained for the *Prp31* alleles (which are in a *w* background), we removed the red pigments of the deficiency lines (caused by the presence of a *w^+^*-minigene) by studying their phenotype in a *cn bw* background, an alternative way to remove all screening pigments. *Df(3L)Exel6262/+*, *Df(3L)ED217/+*, and *Df(3L)ED218/+* flies exhibited retinal degeneration similar as *Prp31^P17^* or *Prp31^P18^* heterozygous flies (Fig. 4), with only about 20% of their ommatidia showing seven rhabdomeres. These deficiency lines also had no obvious effects on retinal development (Fig. S2D–F). Degeneration was also observed in *Prp31^P18^/Df (3L)217* and *Prp31^P17^/Df (3L)217*) flies (Fig. S2G,H).

**Fig. 5.**
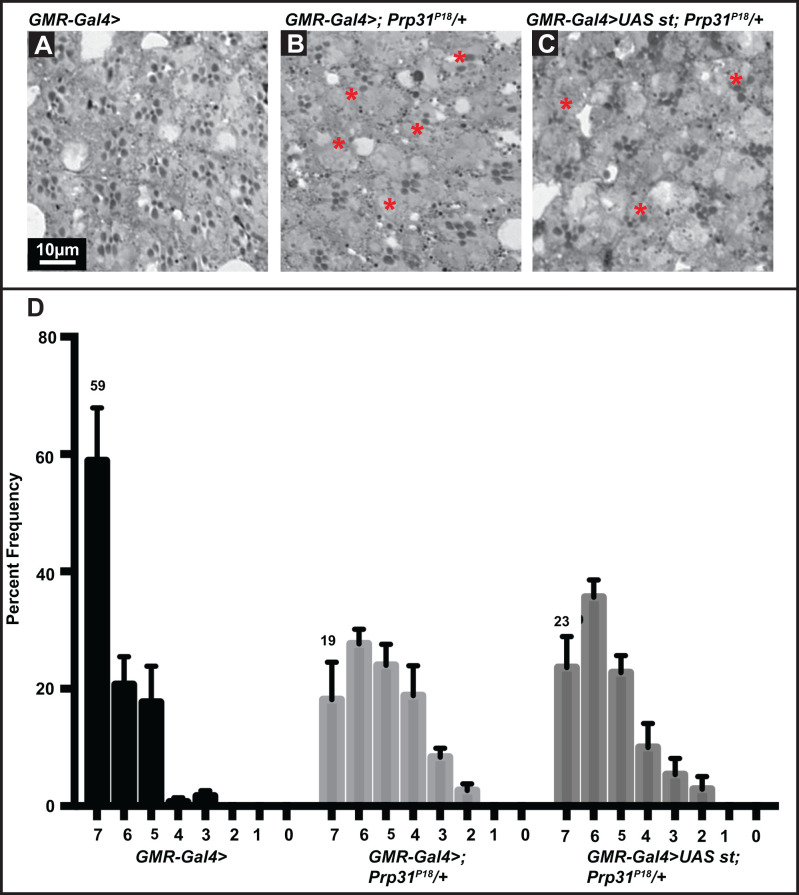
**PRCs of heterozygous *Prp31^P18^* flies undergo light-dependent degeneration in the presence of *st* overexpression.** (A–C) Representative bright-field images of Toluidine-blue stained semi-thin sections of eyes of *GMR-Gal4>* (A; control) and *GMR-Gal4>*; *Prp31^P18^ /+* (B; mutant control) and *GMR-Gal4 >;UAS-st* ; *Prp31^P18^ /+* (C; mutant and *st* overexpression). Complete genotypes can be found in Table S1. Upon eclosion, flies were kept for 2 days under regular light conditions and then subjected to a degeneration paradigm of 7 days of continuous, high-intensity light exposure. In the presence of *st* overexpression, PRCs of heterozygous *Prp31^P18^* flies undergo degeneration to the same extent (C) as without *st* overexpression (B). Scale bar, 10 µm. (D) Quantification of retinal degeneration as indicated by the number of surviving rhabdomeres observed upon high intensity, continuous light exposure. Y-axis: percent frequency of ommatidia displaying one to seven rhabdomeres. Genotypes are indicated below. Number on top of each graph indicates the percentage of the mean number of ommatidia displaying the full complement of seven rhabdomeres. Bars represent mean±s.e.m. (a minimum of *n*=60 ommatidia from eyes of three biological replicates). Statistical significance of differences in this parameter between genotype pairs is indicated in Table S2.

We noticed that retinal degeneration in w*; st^1^/+ flies was enhanced compared to that of w* flies (Fig. 2A,B and E). To rule out that retinal degeneration observed in *Prp31* mutant flies is influenced by the presence of a mutation in *st* mapping close by (Fig. 1A), we overexpressed *st* in the retina of *Prp31^P18^* flies (simultaneously knocking-down *w* gene activity provided by the transgenes). Expression of *st* did not modify the degree of retinal degeneration of *Prp31* mutants (Fig. 5, Table S2).

Taken together, these data support the conclusion that reducing the function of the *Prp31* locus causes light-induced retinal degeneration.

### *Prp31* mutant photoreceptor cells show increased rhodopsin accumulation

**Fig. 6.**
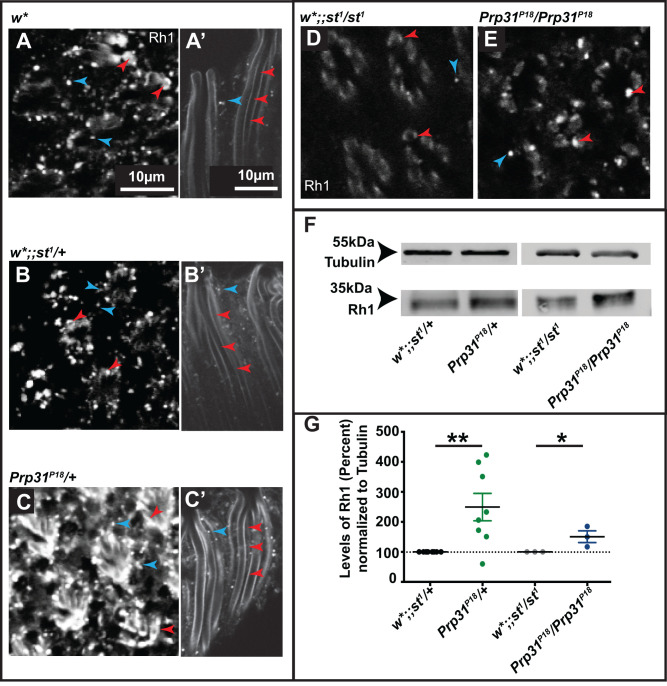
**Reduction of *Prp31* results in increased Rhodopsin 1 accumulation.** Representative confocal images of 1 µm optical sections from 12 µm cross-sections (A–E), or whole mounts (A′–C′) of eyes of adult males with the genotypes indicated, stained with anti-Rh1. Images were taken using the same settings for mutant conditions and their respective controls. Rhabdomeres are shown in cross sections (A–E) and along their length (A′–C′) with the distal end directed towards the top and the proximal end directed towards the bottom. Red arrowheads indicate Rh1 staining in the rhabdomere and blue arrowheads indicate intracellular Rh1. Rh1 staining is more intense in the rhabdomeric membrane of *Prp31^P18^/+* (C,C′) as compared to controls, w* (A,A′) and w*;; st^1^/+ (B,B′). Increased Rh1 immunostaining intensity is also observed in *Prp31^P18^/ Prp31^P18^* (E) as compared to its genetic control (D). Scale bars, 10 µm. (F) Representative western blot for β-Tubulin and Rh1 from head lysates of w*;;Prp31^P18^ /+ and its genetic control w*;;st^1^/+, and for w*;;Prp31^P18^ / Prp31^P18^ and its genetic control w*;;st^1^/ st^1^. Complete genotypes can be found in Table S1. (G) Quantification of Rh1 levels normalised to Tubulin. Graph displays mean±s.e.m. of Rh1 levels calculated from intensity measurements of blots after normalisation compared to that of loading control (Tubulin) with each dot representing one biological replicate. On average, Rh1 levels are increased by 320% (*P*<0.05, Student's Unpaired *t*-test) in w*;;Prp31^P18^/+ as compared to control, w*;;st^1^/+ and by 140% (*P*<0.05, Student's Unpaired *t*-test) in w*;;Prp31^P18^ / Prp31^P18^ as compared to its genetic control w*;;st^1^/ st^1^. Complete genotypes can be found in Table S1.

A common cause of retinal degeneration, both in flies and in mammals, is abnormal localisation/levels of the visual pigment rhodopsin1 (Rh1) (Hollingsworth and Gross, 2012; Xiong and Bellen, 2013). Therefore, we asked if the degeneration observed in *Prp31* mutant retinas is associated with altered Rh1 localisation/levels. *Drosophila* Rh1, encoded by *ninaE*, is the most abundant rhodopsin expressed in the outer PRCs R1-R6 (Harris et al., 1976; Ostroy et al., 1974). In control flies raised under regular light conditions (12 h light, 12 h dark), Rh1 is concentrated in the rhabdomeres. As reported previously, Rh1 either fills the entire rhabdomere, forms a crescent-shaped pattern, or is restricted to the base or the lateral edges of the rhabdomere (Chen et al., 2017; Chinchore et al., 2009; Mitra et al., 2011; Orem et al., 2006; Wang et al., 2014; Xiong et al., 2012). Differences in localisation have been attributed to inconsistencies in antibody penetration due to either the dense packing of microvilli in the rhabdomeres (Xiong et al., 2012) or to a light-induced staining artefact (Schopf et al., 2019). Rh1 staining is observed in rhabdomeres (red arrowheads in Fig. 6A–E) and outlines the rhabdomeric structure along its length (Fig. 6A′–C′). Rh1 could also be detected in cytoplasmic punctae (blue arrowheads in Fig. 6A–E). This intracellular pool of Rh1 presumably represents internalised Rh1 following light exposure (Satoh and Ready, 2005), since these flies were raised under 12 h light/12 h darkness. Strikingly, PRCs of adult flies heterozygous for *Prp31* exhibited increased accumulation of Rh1 in the rhabdomeres in comparison to genetic controls (Fig. 6C,C′). Increased Rh1 immunostaining was observed in mutants independent of light conditions applied (Fig. S3). *Prp31^18^* homozygotes exhibited a similar phenotype of enhanced Rh1 immunostaining intensity (Fig. 6E as compared to D). Further, all three deficiencies that remove the *Prp31* locus exhibited increased Rh1 staining when heterozygous (Fig. S4B–D) in comparison to the genetic controls (Fig. S4A). Finally, RNAi-mediated knockdown of *Prp31* also resulted in increased accumulation of Rh1 immunoreactivity (Fig. S4F) as compared to genetic control (Fig. S4E). Increased intensity of Rh1 immunostaining is due to increased levels of Rh1 as revealed by western blots of protein extracts isolated from adult heads of *Prp31^18^* hetero- and homozygotes. On average, Rh1 levels were significantly increased by over 300% in heads from *Prp31^P18^* heterozygous and by 140% in *Prp31^P18^* homozygous flies as compared to heads of genetic controls (Fig. 6F,G). The variability in the magnitude of increased Rh1 levels (see biological replicates in Fig. 6G) parallels the variability in the degenerative phenotype in the *Prp31* mutants.

### Impaired *Prp31* function does not affect splicing or abundance of *opsin* mRNA, but results in increased *twinfilin* mRNA

To better understand the underlying cause of increased Rh1 in rhabdomeres, we aimed to find out whether *ninaE/opsin1* mRNA levels were altered in these mutants. Using Real time qRT-PCR and primers targeting each of the exons (Table 1A) and the exon-intron junctions (Table 1B), no significant change in *opsin1* mRNA levels was detected in heads of heterozygous and homozygous *Prp31^P18^* flies. This implies that abundance and splicing of *opsin1* mRNA is unaffected in these mutants. We next investigated whether trafficking of opsin/rhodopsin along its biosynthetic route is altered. Carotenoids are precursors of the chromophore 11-cis-3-hydroxyretinal, which binds to opsin to generate the functional visual pigment rhodopsin in flies (von Lintig et al., 2010). Reduction of the chromophore halts endoplasmic reticulum (ER) to Golgi transport and maturation of rhodopsin, resulting in the accumulation of an intermediate form in the perinuclear ER (Colley et al., 1991; Ozaki et al., 1993). Upon supplementation of retinal and induction of its isomerization by blue light, mature Rh1 is now trafficked to the rhabdomere (Satoh et al., 1997). An assay, called blue-light induced chromophore supply (BLICS) (Iwanami et al., 2016; Ozaki et al., 1993), allows to follow Rh1 trafficking along its biosynthetic route. Using the BLICS assay, no qualitative difference was observed in Rh1 reaching the rhabdomere in control and *Prp31^18^* heterozygote flies (Fig. S5). There was no substantial increase in Rh1 reaching the rhabdomere upon its release from the ER. This suggests that the amount of Rh1 produced and trafficked to the rhabdomere (at least via Rab11) was not substantially altered in the *Prp31* mutant.

**Table 1.**
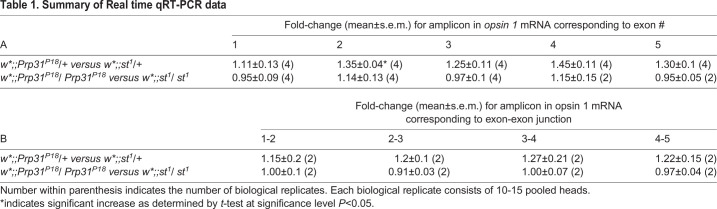
**Summary of Real time qRT-PCR data**

Finally, we evaluated mRNA levels of three genes (Table 2) that have been recently implicated in Rh1 trafficking (Laffafian and Tepass, 2019). Of these, mRNA levels of only *twinfilin* (*twf*), which encodes an actin monomer-binding protein, is increased in *Prp31* mutants (heterozygous and homozygous alleles) as compared to those of the respective genetic background. Taken together, impaired *Prp31* function is associated with increased rhodopsin protein levels and increased *twinfilin* mRNA, but does not affect the amount or splicing of *opsin* mRNA.

**Table 2.**
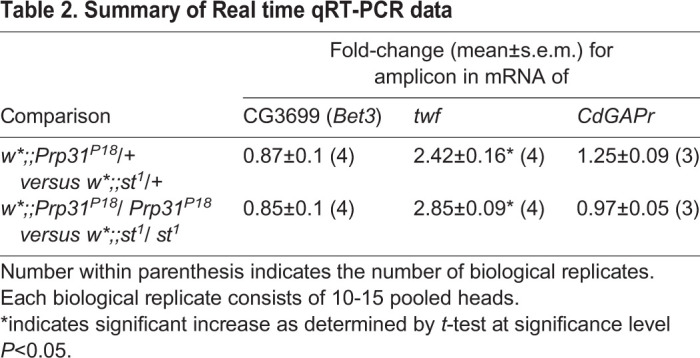
**Summary of Real time qRT-PCR data**

### Light-dependent photoreceptor degeneration in *Prp31* mutants is suppressed upon elimination of Rh1 accumulation

**Fig. 7.**
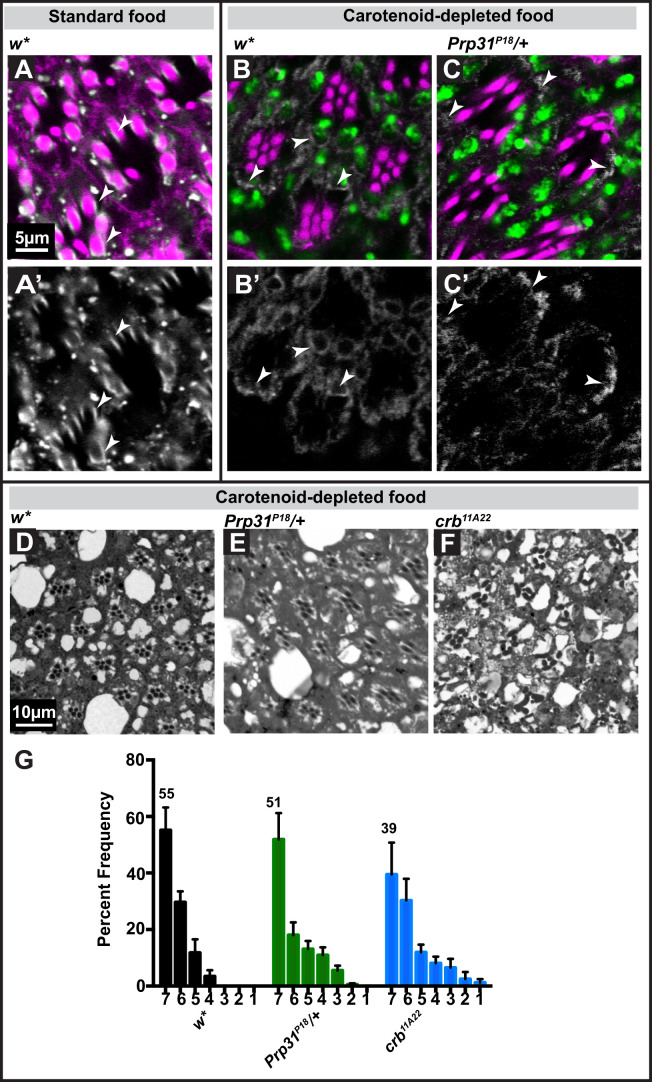
**Carotenoid-depleted diet limits the extent of light-induced degeneration in heterozygous *Prp31* mutants.** (A–C′) Representative images of 1 µm confocal optical sections from 12 µm cryosections of male eyes. Genotypes indicated. Tissues were immunostained for Rh1 (white) and labelled with phalloidin (magenta) and DAPI (green), to stain the rhabdomeres and nuclei, respectively. (A–C) Overlay of two (A) and three (B,C) channels, (A′–C′) images showing the extracted channel (Rh1). Reduction in Rh1 levels and change in its localisation from the rhabdomeres to a peri-nuclear localisation is observed in control (B,B′) and mutant (C,C′) flies fed on a carotenoid-depleted diet (B–C′) as opposed to flies fed on standard food (A–A′). Arrowheads indicate Rh1 localisation in the rhabdomere as opposed to peri-nuclear localisation. Scale bar, 5 µm. (D–F) Representative bright-field images of Toluidine-blue stained semi-thin sections of eyes of w* (D), w*;Prp31^P18^/+ (E) and w*;;crb^11A22^ (F) adults. Complete genotypes can be found in Table S1. Animals were raised on a carotenoid-depleted diet. Upon eclosion, they were aged for 2 days under regular light conditions and then subjected to a degeneration paradigm of exposure for 7 days to continuous, high-intensity light. Scale bar, 10 µm. (G) Quantification of retinal degeneration as indicated by the number of surviving rhabdomeres observed upon high intensity, continuous light exposure. Y-axis: percent frequency of ommatidia displaying one to seven rhabdomeres. Genotypes are indicated below. Number on top of each graph indicates the mean percentage of ommatidia displaying the full complement of seven rhabdomeres. Bars represent mean±s.e.m. (a minimum of *n*=60 ommatidia from eyes of three biological replicates). Statistical significance of differences in this parameter between genotype pairs is indicated in Table S2.

There is evidence suggesting that increased levels of rhodopsin and/or mis-localised rhodopsin contributes to retinal degeneration, both in mammals and in flies (Hollingsworth and Gross, 2012). In *crb* mutant retinas, for example, the degree of light-dependent retinal degeneration could be strongly reduced in animals raised on carotenoid-depleted food (Johnson et al., 2002). To determine whether rhodopsin accumulation makes the retina of *Prp31* mutant flies prone to light-induced degeneration, we experimentally reduced rhodopsin levels by raising animals in carotenoid-free diet from embryonic stages onward. In fact, in retinas of flies raised under this condition, Rh1 levels are reduced and rhabdomeric localisation of Rh1 is abolished. Instead, Rh1 can now be found perinuclear, both in control and in *Prp31/+* retinas (Fig. 7B–C′). In contrast, in the retina of control flies raised on normal food, Rh1 is detected on the lateral edges of the rhabdomeres (Fig. 7A,A′, white arrowheads).

After 7 days of constant light exposure, the overall appearance of the retinae of both the genetic control (w*) and *Prp31/+* mutants appeared more damaged as revealed by fewer surviving rhabdomeres and more lacunae (compare the retina of w* in Figs 2 and 7). Note that rhabdomeres are smaller under this dietary condition, a result which is consistent with previous reports (Johnson et al., 2002; Sapp et al., 1991; Satoh et al., 1998). More importantly, the percentage of ommatidia with seven rhabdomeres was the same in heterozygous *Prp31^P18^/+* and control (w*) retinas under carotenoid depletion (Fig. 7D,E and G). A similar result was obtained in *crb* mutant retinas prepared from flies raised under the same conditions (Fig. 7F,G) (Johnson et al., 2002), supporting the conclusion that carotenoid depletion prevents retinal degeneration.

To conclude, these results suggest that Rh1 accumulation in *Prp31* mutant flies makes the retina more susceptible to light-induced degeneration.

## DISCUSSION

Here we present a fly model for RP11, an autosomal-dominant human disease caused by mutations in the splicing regulator PRPF31, which leads to blindness in affected patients. Our results reveal that mutations in the *Drosophila* orthologue *Prp31* induce PRC degeneration under light stress, thus mimicking features of RP11-associated symptoms. Similar to those in human, mutations in *Drosophila Prp31* are haplo-insufficient and lead to retinal degeneration when heterozygous. This is in stark contrast to mice heterozygous for *Prpf31*, which did not show any signs of PRC degeneration (Bujakowska et al., 2009), but rather late-onset defects in the retinal pigment epithelium (Farkas et al., 2014; Graziotto et al., 2011).

By using three different genetic approaches we provide convincing evidence that the knockdown of *Prp31* is the cause of the retinal degeneration observed. (1) The two *Prp31* alleles induced by TILLING (*Prp31^P17^* and *Prp31^P18^*) carry missense mutations in conserved amino acids of the coding region, which are predicted to be damaging. (2) Flies heterozygous for any of three deletions, which completely remove the *Prp31* locus, exhibit comparable phenotypes as flies heterozygous for *Prp31* point mutations. (3) RNAi-mediated knockdown of *Prp31* results in light-induced retinal degeneration. The results obtained suggest that the two missense mutations mapped in *Prp31^P17^* and *Prp31^P18^* are strong hypomorphic alleles. First, the two *Drosophila* alleles characterised here are hemizygous (*Prp31*/deficiency) and homozygous (in the case of *Prp31^P18^*) viable and fertile. Second, mutations in the two established *Prp31* fly lines are missense mutations, one located N-terminal to the NOSIC domain in *Prp31^P17^* (G90R) and the other in the Nop domain in *Prp31^P18^* (P277L) (see Fig. 1A), which most likely result in a reduced function of the respective proteins (Fig. S1B). Whether protein levels are also decreased cannot be answered due to the lack of specific antibodies. The mutated amino acid residue in *Drosophila Prp31^P18^* (P277L) lies within the NOP domain. Interestingly, many point mutations in human PRPF31, which are linked to RP11, have been mapped to the Nop domain (Liu and Zack, 2013; Vithana et al., 2001; Wheway et al., 2020) (see ClinVar: https://www.ncbi.nlm.nih.gov/clinvar/?term=PRPF31%5Bgene%5D). Similar as in yeast (Hardin et al., 2015), the Nop domain in human PRPF31 is involved in an essential step in the formation of the U4/U6-U5 tri-snRNP by building a complex of the U4 snRNA and a 15.5K protein, thus stabilising the U4/U6 snRNA junction. The mutated proline in *Drosophila Prp31^P18^* precedes a histidine (H278), which corresponds to amino acid H270 in the human protein (see Fig. S1A). Mutations in H270 in the Nop domain of human PRPF31 result in a reduced affinity of PRPF31 to the complex formed by a stem-loop structure of the U4 snRNA and the 15.5K protein (Liu et al., 2007; Schultz et al., 2006). Therefore, it is tempting to speculate that the *Drosophila* P277L mutation could similarly weaken, but not abolish the corresponding interaction of the mutant Prp31 protein with the U4/U6 complex. Further experiments are required to determine the functional consequences of the molecular lesions identified in *Drosophila* PRP31.

We noticed that the retinal phenotype observed upon reduction of *Prp31* is more variable than that observed upon loss of *crb* (see, for example, Fig. 2E) (Johnson et al., 2002; Spannl et al., 2017). This could be due to the fact that all *Prp31* conditions analysed represent hypomorphic conditions, possibly retaining some residual protein function(s). However, the expressivity of the mutant phenotype is not increased in *Prp31*/deficiency flies (carrying only one mutant copy) in comparison to that of *Prp31/+* flies, which carry one mutant and one wild-type allele. Interestingly, human RP11 patients heterozygous for mutations in *Prpf31* show an unusually high degree of phenotypic non-penetrance and can even be asymptomatic. Various causes have been uncovered to explain this feature (Wheway et al., 2020). These include a highly variable expression level of the remaining wild-type *Prpf31* allele, possibly due to changes in the expression levels of trans-acting regulators (Rio Frio et al., 2008) (reviewed in Rose and Bhattacharya, 2016). In addition, mutant PRPF31 proteins can form cytoplasmic aggregates in RPE cells, thus reducing the amount of protein entering the nucleus (Valdes-Sanchez et al., 2019), or can impair overall transcription or splicing, as described in *Prpf31* zebrafish models (Linder et al., 2011; Yin et al., 2011). Finally, mutations in unlinked genes have been suggested to modify the disease severity of patients (Venturini et al., 2012).

Not only in flies, but also in human, mutations in *PRPF31* affect only the retina, despite the importance of this splicing regulator in all cells. Recently published data show that impaired *PRPF31* function can affect the splicing of target genes in a cell-type specific manner. Strikingly, retinal cells isolated from RP11 patient-derived retinal organoids exhibit mis-splicing of genes that encode components of the splicing machinery itself. This was not observed in fibroblasts or iPS cells derived from the same patients (Buskin et al., 2018). These authors obtained similar results from the retina and the RPE of *Prpf31/+* mice. Mutant RPE cells additionally revealed splicing defects in transcripts of genes with functions in ciliogenesis, cell polarity and cellular adhesion (Buskin et al., 2018), which could explain the previously described RPE defects in these mice (Farkas et al., 2014; Graziotto et al., 2011; Hamieh and Nandrot, 2019).

In the retina of flies lacking one functional copy of *Prp31*, PRCs showed increased levels of Rh1, both in the rhabdomeres and in the cytoplasm, as revealed by immunostaining and confirmed by western blot analysis. However, increased Rh1 levels did not affect rhabdomere size or structure. This is in contrast to results obtained in the mouse, where transgenic overexpression of either wild-type bovine or human rhodopsin induced an increase in outer segment volume of rod PRCs (Price et al., 2012; Wen et al., 2009). In several other *Drosophila* mutants, accumulation of Rh1 in endocytic compartments has been suggested to cause retinal degeneration due to its toxicity. For example, dominant mutations in *Drosophila ninaE* result in ER accumulation of misfolded Rh1 due to impaired protein maturation. This, in turn, causes an overproduction of ER cisternae and induces the unfolded protein response (UPR), which eventually leads to apoptosis of PRCs, both in flies and in mammals (Colley et al., 1995; Kroeger et al., 2018; Zhang et al., 2014).

Interestingly, mis-localisation of rhodopsin in human PRCs to sites other than the outer segment is a common characteristic of adRP induced by mutations in rhodopsin and is considered to contribute to the pathological severity (Hollingsworth and Gross, 2012). Our data suggest that increased accumulation of rhodopsin contributes to degeneration in *Prp31* mutant retinas. Reduction of Rh1 by depletion of dietary carotenoid not only obliterated increased Rh1 immunoreactivity and opsin retention in perinuclear compartments in *Prp31* mutants, but also reduced the degree of PRC degeneration. However, whether increased Rh1 accumulation in the rhabdomere or in the cytoplasm contributes to light-dependent PRC degeneration of *Prp31* mutant flies needs to be explored in the future.

Our data further suggest that *Prp31* regulates, directly or indirectly, Rh1 levels at a posttranscriptional level, since no increase of RNA levels was detected in heads of *Prp31/+* flies. This result is different from that obtained in primary murine retinal cell cultures, where expression of a mutant *Prpf31* gene reduced rhodopsin expression, as a result of impaired splicing of the rhodopsin pre-mRNA (Yuan et al., 2005). Similarly, siRNA-mediated knockdown of *PRPF31* function in human organotypic retinal flat-mount cultures (HORFC) reduced mRNAs encoding genes involved in phototransduction and photoreceptor structure, including rhodopsin (Azizzadeh Pormehr et al., 2018). Interestingly, the *Prp31* mutants described here show increased mRNA levels of an evolutionary conserved actin monomer binding protein called *twinfilin* (*twf*), which inhibits actin polymerisation. Knockdown of *twf* results in excessive cytoplasmic Rh1 staining, suggesting defects in its trafficking (Laffafian and Tepass, 2019). In *Prp31* mutants, an increase in rhabdomeric Rh1 was observed as well as increased *twf* mRNA. From this correlation we hypothesise that upregulation of *twf* mRNA in *Prp31* might be in part responsible for at least the rhabdomeric Rh1 accumulation. Rh1 also accumulates in the cytoplasm of *Prp31* mutant PRCs. Our data exclude the role of Rab11-mediated targeting of Rh1 in this accumulation. Now, it remains to be determined if the deregulation of other trafficking routes or the upregulation of *twf* contributes to the increased Rh1 in the cytoplasm. In the future, it may be interesting to explore the link between increased Rh1 levels as observed in *Drosophila Prp31* mutants, increased mRNA levels of *twinfilin* and impaired Rh1 trafficking. Additionally, a detailed transcriptome analysis should elucidate possible defects in transcription and/or splicing of target genes, thus also allowing a better understanding of the aetiology of the human disease.

## MATERIALS AND METHODS

### Fly strains and genetics

All phenotypic analyses were performed in age-matched males unless otherwise specified. Genotypes are summarised in Table S1. Flies were maintained at 25°C on standard yeast-cornmeal-agar food unless otherwise stated. To rule out differences in light sensitivity in the light-degeneration paradigm, we used white-eyed flies, bearing mutations in the *white* gene, both as general controls and in the respective mutant background. The *white* allele (w*) used here was tested by PCR and shown to carry a deletion that includes the transcription and translation start site of the *white* gene (data not provided). Loss of *scarlet* (*st*) function was rescued by Gal4-mediated expression of a *scarlet* transgene (Cunningham et al., 2018) in all cells of the retina using GMR-Gal4 (Hay et al., 1994). The RNAi line (ID: 35131) for the *Prp31* gene was obtained from the Vienna *Drosophila* Resource Centre (VDRC, www.vdrc.at) (Dietzl et al., 2007). RNAi was induced using *Rh1-Gal4* (Lee and Carthew, 2003) in combination with Dicer-2 expression and concomitant expression of *white* RNAi under the control of the GMR-promoter (*GMR-w^IR^*) (Lee and Carthew, 2003) allowing assay of degeneration in a non-pigmented background. *Df(3L)Exel6262* with deleted segment 71B3;71C1 (Parks et al., 2004)*, Df(3L)ED217* with deleted segment 70F4;71E1 and *Df(3L)ED218* with deleted segment 71B1–71E1 (Ryder et al., 2007) were obtained from the Bloomington Stock Centre. Since the deficiency lines carry a mini-*white* transgene due the way they were generated (Ryder et al., 2007), *cn bw* was recombined into these lines to obtain white-eyed flies and all phenotypes were compared with *cn bw*.

### Isolation of *Prp31* alleles by TILLING

To isolate point mutations in the *Prp31* locus (FlyBase ID: FBgn0036487) a library of 2.400 fly lines with isogenised third chromosomes, which potentially carry point mutations caused by EMS treatment, was screened (Winkler et al., 2005). Our approach targeted exon 1–3 of the *Prp31* locus containing two thirds (67%) of the coding sequence, which includes several predicted functional domains (the NOSIC (IPRO012976), the Nop (IPRO002687) and parts of the Prp31_C terminal (IPRO019175) domain), making use of two different PCR amplicons. A nested PCR approach was used, where the inner primers contain universal M13 tails that serve as primer binding sites of the Sanger sequencing reaction:

- amplicon1 (covers exon 1 and 2), outer primer, forward: TTCAATGAACCGCATGG, reverse: GTCGATCTTTGCCTTCTCC, inner / nested primer, forward: TGTAAAACGA CGGCCAGT-AGCAACGGTCACTTCAATTC, reverse: AGGAAACAGCTATGACCAT-GAAAGGGAATGGGATTCAG);
- amplicon 2 (covers exon 3), outer primer, forward: ATCGTGGGTGAAATCGAG, reverse: TGGTCTTCTCATCCACCTG, inner / nested primer, forward: TGTAAAACGA CGGCCAGT-AAGCTGCAGGCTATTCTCAC, reverse: AGGAAACAGCTATGACCAT-TAGGCATCCTCTTCGATCTG.

PCR-reactions were performed in 10 µl volume and with an annealing temperature of 57°C, in 384-well format, making use of automated liquid handling tools. PCR fragments were sequenced by Sanger sequencing optimised for amplicon re-sequencing in a large-scale format (Winkler et al., 2011, 2005). Primary hits, resembling sequence variants, which result in potential nonsense and missense mutations upon translation or affect a predicted splice site, were verified in independent PCR amplification and Sanger sequencing reactions. Two of the four lines, named *Prp31^P17^* and *Prp31^P18^*, were recovered from the living fly library and crossed for three generations to control, w* flies to reduce the number of accompanying sequence variations. The removal of the markers of the original, mutagenised chromosome (*ru st e ca*) by the above outcrossing was verified as follows: the isolated alleles (males) were crossed to the original line (*ru st e ca*) and checked for the phenotypes associated with homozygous conditions of *roughoid* (*ru*; eye appearance)*, scarlet* (*st*; eye colour)*, ebony* (*e*; body colour)*, claret* (*ca*; eye colour)*.*

### Experimental light conditions

Flies were reared in regular light conditions defined as 12 h of light (approximately 900–1300 lux)/12 h of darkness. For the light-induced degeneration setting, flies (2 days of age) were placed at 25°C for 7 days in an incubator dedicated for continuous, high intensity light exposure (Johnson et al., 2002). High intensity light was defined by 1200–1300 lux measured using an Extech 403125 Light ProbeMeter (Extech Instruments, USA) with the detector placed immediately adjacent to the vial and facing the nearest light source. At the end of 7 days, fly heads were processed for sectioning. For immunostaining and western blotting, flies (1 day) reared under regular light were processed as described below.

### Vitamin A depletion, BLICS assay

For vitamin A depletion experiments, animals were raised and maintained from embryonic stages onward on carotenoid free food (10% dry yeast, 10% sucrose, 0.02% cholesterol, and 2% agar) as described (Pocha et al., 2011). The protocol for BLICS assays was as described in Hebbar et al., 2020 doi: https://doi.org/10.1083/jcb.201911100). Briefly, flies were raised on carotenoid free food and upon eclosion, they were placed in the dark containing carotenoid-free food or food supplemented with all-trans retinal. After 48 h, flies were exposed to blue light and after 180 min, fly heads were quickly dissected and put in fixative for cryosectioning and immunostaining. At least two regions of interest (comprising at least 6–8 ommatidia) from three biological replicates (fly eyes) were used for quantification.

### Quantification of Degeneration

Toluidine blue stained semi-thin sections were imaged with a 63x Plan Apo oil objective (N.A. =1.4) on AxioImager.Z1 (Zeiss, Germany), fitted with AxioCamMRm camera, and analysed using the AxioVision software (Release 4.7). Quantification of degeneration was performed as described (Bulgakova et al., 2010). Briefly, the number of detectable rhabdomeres in each ommatidium was recorded from approximately 60–80 ommatidia per section and at least three eyes from different individuals were analysed. In case of degeneration, fewer ommatidia were counted since most of the tissue had degenerated.

### Immunostaining of adult retina and confocal imaging

Adult eyes were dissected and fixed in 4% formaldehyde. They were then processed either directly for immunostaining of the whole eye after removal of the lens, or for cryosectioning. For sectioning, sucrose treatment and embedding of the tissues in Richard-Allan Scientific NEG-50^TM^ (Thermo Fisher Scientific, UK) tissue embedding medium was done (Mishra and Knust, 2019). Eyes were cryosectioned at 12 µm thickness at -21°C. Sections were air-dried and then subjected to immunostaining, which was done as described previously (Spannl et al., 2017). Antibodies used were mouse anti-Rh 1 (1:50; 4C5) from Developmental Studies Hybridoma Bank (DSHB), University of Iowa, IA, USA. 4C5 [http://dshb.biology.uiowa.edu/4C5] was deposited to the DSHB by de Coet, H.G./Tanimura, T., and by Fambrough, D.M., and anti-Rab11 (1:1000; Satoh et al., 2005; kind gift of D. Ready). Alexa-Fluor conjugated secondary antibodies (1:200, Thermo Fisher Scientific, UK) were used. DAPI (4′,6-Diamidino-2-Phenylindole, Dihydrochloride; Thermo Fisher Scientific, UK) was used to label nuclei in tissue sections and Alexa-Fluor-555–phalloidin (Thermo Fisher Scientific, UK) was used to visualise F-actin enriched rhabdomeres. Sections and whole mounts were imaged with an Olympus Fluoview 1000 confocal microscope using an Olympus UPlanSApochromat 60x Oil objective (N.A. =1.35). They were subsequently visualised in Fiji (Schindelin et al., 2012) and corrected for brightness and contrast only.

### Western blotting

Five fly heads from each genotype were homogenised in 10 µl of 4× SDS-PAGE sample buffer (200 mM Tris-HCl pH 6.8, 20% Glycerol, 8% SDS, 0.04% Bromophenol blue, 400 mM DTT). After dilution with RIPA buffer (150 mM sodium chloride, 1% Triton X-100, 0.5% sodium deoxycholate, 0.1% SDS, 50 mM Tris pH 8), lysates were heated at 37°C for 30 min. Lysates equivalent to 2.5 heads were loaded and run on a 15% acrylamide gel, and proteins were transferred onto a membrane (Nitrocellulose Blotting Membrane 10600002; GE Healthcare Life Sciences, PA, USA). Primary antibodies were incubated overnight at 4°C and included (1) anti-Rh1 [4C5 (http://dshb.biology.uiowa.edu/4C5); 1:500], from Developmental Studies Hybridoma Bank (DSHB), University of Iowa, IA, USA, deposited by de Coet, H.G./Tanimura, T. and (2) anti-α-Tubulin (T6074; 1:5000) from Sigma-Aldrich. As secondary antibody IRDye 800CW goat anti-Mouse IgG (1:15,000; LI-COR Biotechnology, NE, USA) was used for a 1 h incubation at room temperature. The fluorescent signal from the dry membrane was measured using a LI-COR Odyssey Sa Infrared Imaging System 9260-11P (LI-COR Biotechnology). The intensity of the bands was analysed using the Image Studio Ver 4.0 software. The reported value in Fig. 6 was obtained following normalisation of the intensity values for Rh1 with the corresponding Tubulin intensity values and the number of heads loaded onto the gel.

### Real Time qRT-PCR analyses

RNA extraction, cDNA generation, qPCR analyses were performed as described in Hebbar et al. (2020) (doi: https://doi.org/10.1083/jcb.201911100). Primers are listed in Table S3.

### Figure panel preparation

All figure panels were assembled using Adobe Photoshop CS5.1 or Adobe Illustrator CS3 (Adobe Systems, USA). Statistical analyses and graphs were generated using GraphPad Prism (GraphPad Software, Inc, USA) and Microsoft Excel. For protein sequence visualisation, Illustrator of Biological Sequences (IBS; Liu et al., 2015) software package was used.

## Supplementary Material

Supplementary information
