# Supplementary material for: Mutations in the splicing regulator Prp31 lead to retinal degeneration in Drosophila
Source: Biol Open. 2021 Jan 25;10(1):bio052332. doi: 10.1242/bio.052332 (PMC7860132; doi:10.1242/bio.052332)
Supplement: Supplementary information [file biolopen-10-052332-s1.pdf]

A

```
D.m.      MSLADELLADLEEDNDNELEEEDSEMASAEDES--LLAEKLAKPAPNLMDVDV-TVQSVR   57
D.r.      MSLADELLADLEEAGEEDGLYPGGEEGESDGEPGERQVDGGLEDIPEEMEVDYSSTESVT   60
H.s.      MSLADELLADLEEAAEEEE---GGSYG-------EEEEEPAIEDVQEETQLDLS-GDSVK   49
M.m.      MSLADELLADLEEAAEEEE---GGSYG-------EEEEEPAIEDVQEETQLDLS-GDSVK   49
          *************  :::    ... .           :   :   :  ::*    :**

                                           G→R
D.m.      ELCKLRDSERLKNTLQQIEHYASRQRTAAEMLGSVESDPEYCLIVDANAIAVDIDNEISI   117
D.r.      SIAKLRHSKPFAEIMDKISHYVGNQRKNSEVSGPVEADPEYRLIVAANNLTVEIDNELNI   120
H.s.      TIAKLWDSKMFAEIMMKIEEYISKQAKASEVMGPVEAAPEYRVIVDANNLTVEIENELNI   109
M.m.      SIAKLWDSKMFAEIMMKIEEYISKQANASEVMGPVEAAPEYRVIVDANNLTVEIENELNI   109
           :.** .*: : : : :*..* ..* . :*: * **: *** :** ** ::*:*:**:.*

D.m.      VHKFTKEKYQKRFPELDSLIVGEIEYLLAVKELGNDLDQVKNNEKLQAILTQATIMIVSV   177
D.r.      IHKFVRDKYSKRFPELESLVPNALDYIRTVKELGNNLEKCKNNETLQQILTNATIMVVSV   180
H.s.      IHKFIRDKYSKRFPELESLVPNALDYIRTVKELGNSLDKCKNNENLQQILTNATIMVVSV   169
M.m.      IHKFIRDKYSKRFPELESLVPNALDYIRTVKELGNSLDKCKNNENLQQILTNATIMVVSV   169
          :*** ::**.******:**: . ::*: :******.*:: ****.** ***:****:***

D.m.      TASTTQGTMLTPAEKAKIDEACEMAIELNNFKSKIYEYVESRMTFIAPNLSMIVGASTAA   237
D.r.      TASTTQGTMLGDDELQRLEEACDMALELNQSKHRIYEYVESRMSFIAPNLSIIVGASTAA   240
H.s.      TASTTQGQQLSEEELERLEEACDMALELNASKHRIYEYVESRMSFIAPNLSIIIGASTAA   229
M.m.      TASTTQGQQLSDEELERLEEACDMALELNASKHRIYEYVESRMSFIAPNLSIIIGASTAA   229
          *******  *   *  :::***:**:***  * :*********:*******:*:******

                                                        P→L
D.m.      KLLGIAGGLSKLSKMPACNVQVLGAQKKTLSGFSQTQMLPHTGYVYYSQIVQDTAPDLRR   297
D.r.      KIMGVAGGLTNLSKMPACNLMLLGAQRRTLSGFSSTSLLPHTGYIYHCDVVQTLPPDLRR   300
H.s.      KIMGVAGGLTNLSKMPACNIMLLGAQRKTLSGFSSTSVLPHTGYIYHSDIVQSLPPDLRR   289
M.m.      KIMGVAGGLTNLSKMPACNIMLLGAQRKTLSGFSSTSVLPHTGYIYHSDIVQSLPPDLRR   289
          *::*:****::*********: :****::******.*.:******:*:.::**  *****

D.m.      KAARLVAAKSVLAARVDACHESVHGEIGLRFKEDVEKKLDKLQEPPPVKFIKPLPKPIEG   357
D.r.      KAARLVSAKCTLASRVDSFHESADGKVGYDLKEEIERKFDKWQEPPPVKQVKPLPAPLDG   360
H.s.      KAARLVAAKCTLAARVDSFHESTEGKVGYELKDEIERKFDKWQEPPPVKQVKPLPAPLDG   349
M.m.      KAARLVAAKCTLAARVDSFHESTEGKVGYELKDEIERKFDKWQEPPPVKQVKPLPAPLDG   349
          ******:**..**:***: ***..*::*  :*:::*:*:** ******* :**** *::*

D.m.      SKKKRGGKRVRKMKERYALTEFRKQANRMNFGDIEEDAYQGDLGYSRGTIGKTGTGRIRL   417
D.r.      QRKKRGGRRYRKMKERLGLTEIRKHANRMTFAEIEDDAYQEDLGFSLGQLGKSGSGRVRQ   420
H.s.      QRKKRGGRRYRKMKERLGLTEIRKQANRMSFGEIEEDAYQEDLGFSLGHLGKSGSGRVRQ   409
M.m.      QRKKRGGRRYRKMKERLGLTEIRKQANRMSFGEIEEDAYQEDLGFSLGHLGKSGSGRVRQ   409
          .:*****:* ****** .***:**:****.*.:**:**** ***:* * :**:*:**:*

D.m.      PQVDEKTKVRISKTLHKNLQKQQ-VYGGNTTVKRQISGTASSVAFTPLQGLEIVNPQAAE   476
D.r.      AQVNDSTKARISKSLQRTLQKQSMTYGGKSTVRDRSSGTSSSVAFTPLQGLEIVNPQAAE   480
H.s.      TQVNEATKARISKTLQRTLQKQSVVYGGKSTIRDRSSGTASSVAFTPLQGLEIVNPQAAE   469
M.m.      TQVNEATKARISKTLQRTLQKQSVVYGGKSTIRDRSSGTASSVAFTPLQGLEIVNPQAAE   469
           **:: **.****:*::.****. .***::*:: : ***:********************

D.m.      RSQTEANAKYFSNTSGFMSVGQRTT-----   501
D.r.      KKVAEANQKYFSNMAEFLKVKREKEDKV--   508
H.s.      KKVAEANQKYFSSMAEFLKVKGEKSGLMST   499
M.m.      KKVAEANQKYFSSMAEFLKVKGEKSGTMST   499
          :. :*** ****. : *:.*  ..
```

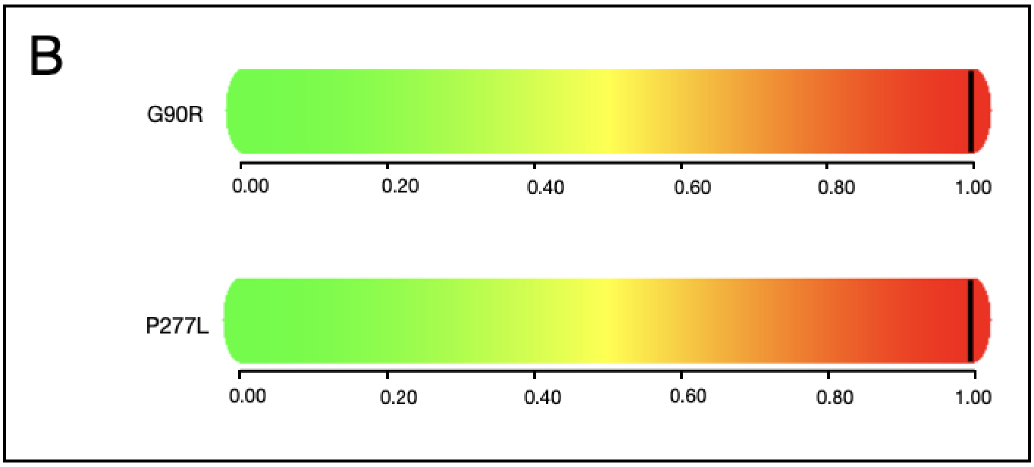

Biology Open • Supplementary information

Figure S1: Molecular defects in *Prp31* mutant flies

A. Amino acid sequence alignment of Prp31 proteins of *Drosophila melanogaster* (D. m.), *Danio rerio* (D. e.), *Homo sapiens* (H. s.) and *Mus musculus* (M. m.) The NOSIC (yellow), Nop (red) and Prp31_C specific (green) domains are indicated as described on UniProt and Pfam websites. Asterisks (*) indicate identical amino acids, colons (:) indicate conserved amino acids, (.) indicate amino acids with weak conservation. Alignment was made using ClustalO

1.2.3. The two TILLING mutations (G82R and P269L) are highlighted in blue.

B. PolyPhen-2 score predictions for the two *Prp31* variants. The G82R mutation is predicted to be probably damaging with a score of 0.978, the P277L mutation is predicted to be probably damaging with a score of 0.981.

Biology Open • Supplementary information

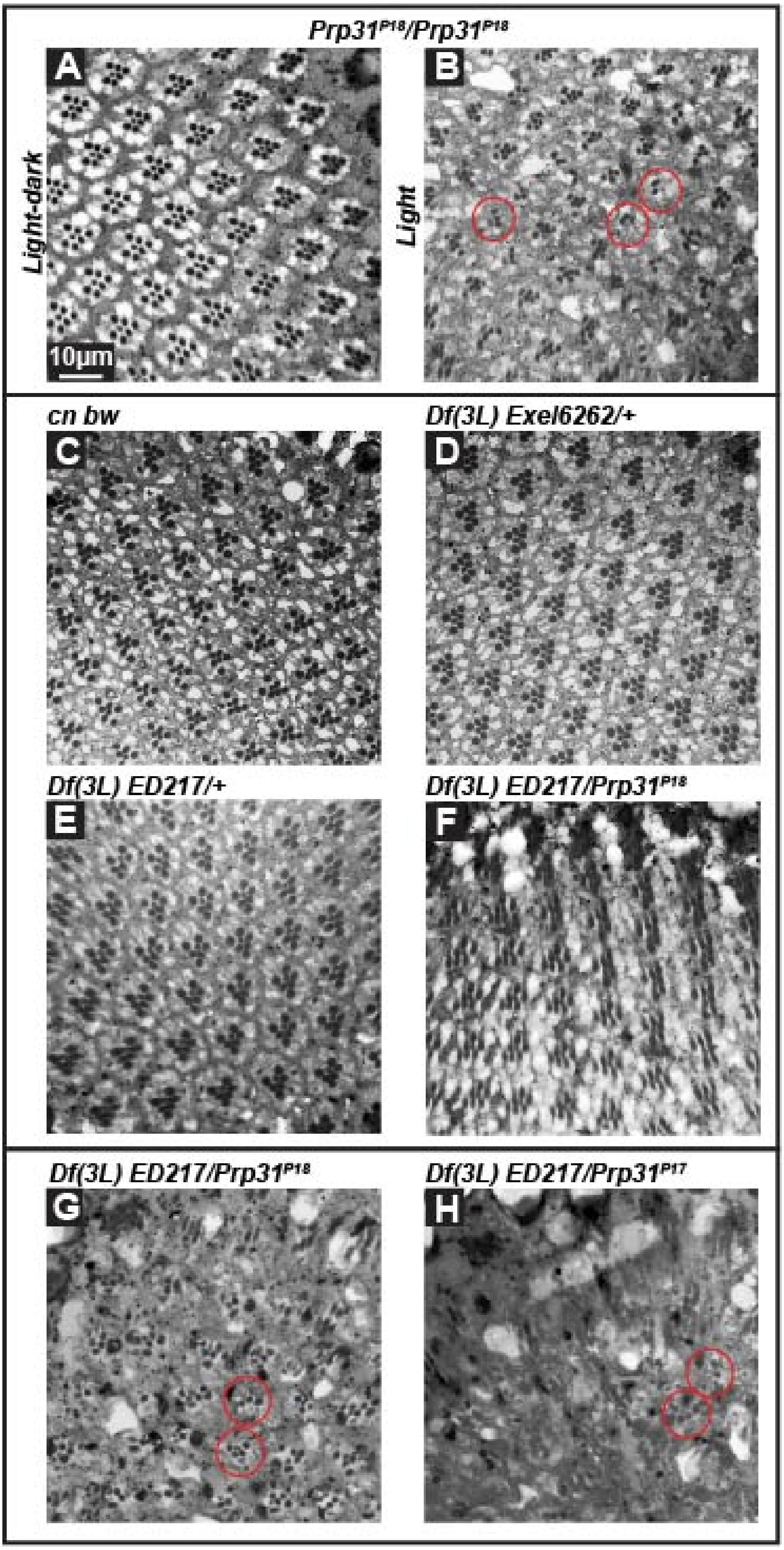

Figure S2

Biology Open • Supplementary information

Figure S2: Homozygous and hemizygous *Prp31* mutants exhibit normal retinal development but display light-induced retinal degeneration.

(A-H) Representative bright-field images of Toluidine-blue stained, semi-thin, sections of eyes of homozygous (A-B), and hemizygous (D-H) *Prp31* mutants reared under light conditions as specified below. Complete genotypes can be found in Suppl. Table S1. Scale bar = 10 µm (A-B). Upon eclosion, *Prp31*^{P18}/*Prp31*^{P18} males were kept for two days under regular light conditions. Eyes were either processed immediately for imaging (A), or after subjecting flies to a degeneration paradigm of 7 days exposure to continuous, high intensity light (B). Red circles outline individual ommatidia. (C-D) Upon eclosion, males of *cn bw* (C), *Df (3L) Exel 6262/+* (D), *Df (3L) ED217/+* (E) and *Df (3L) ED217/Prp31*^{P18} (F) were kept for 2 days under regular light conditions. (G-H) Upon eclosion, males of *Df(3L)ED217/Prp31*^{P18} (G) and *Df(3L)ED217/Prp31*^{P18} (H), were subjected to a degeneration paradigm of 7 days exposure to continuous, high intensity light. Red circles outline individual ommatidia.

Biology Open • Supplementary information

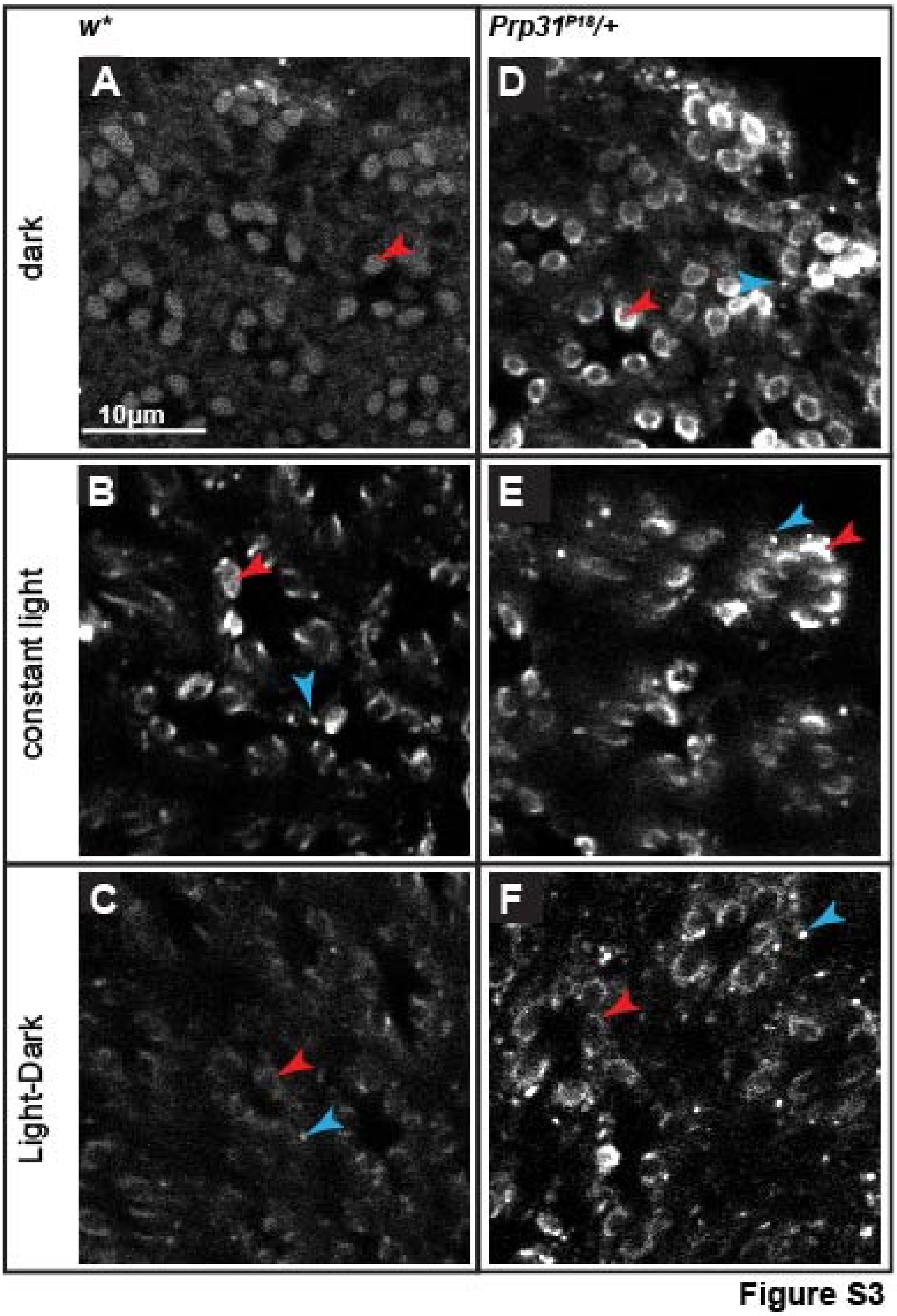

Figure S3: Rh1 immunostaining pattern in eyes of flies reared under different light conditions

A-F are confocal images of 1µm optical sections from 12µm cross-sections of eyes of 2 days old adults reared under constant darkness (A, D), constant light (B, E) and 12h light/12h darkness (C, F). *w** (A-C) and *Prp31*^{P18}/+ (D-F) retinas were stained with anti-Rh1.

Red arrowheads indicate rhabdomeric staining, blue arrowheads indicate intracellular Rh1 labelling. Note increased Rh1 intensity in mutant retinas under all light conditions. Images were taken using the same settings as their respective controls. Scale bar = 10 µm.

Biology Open • Supplementary information

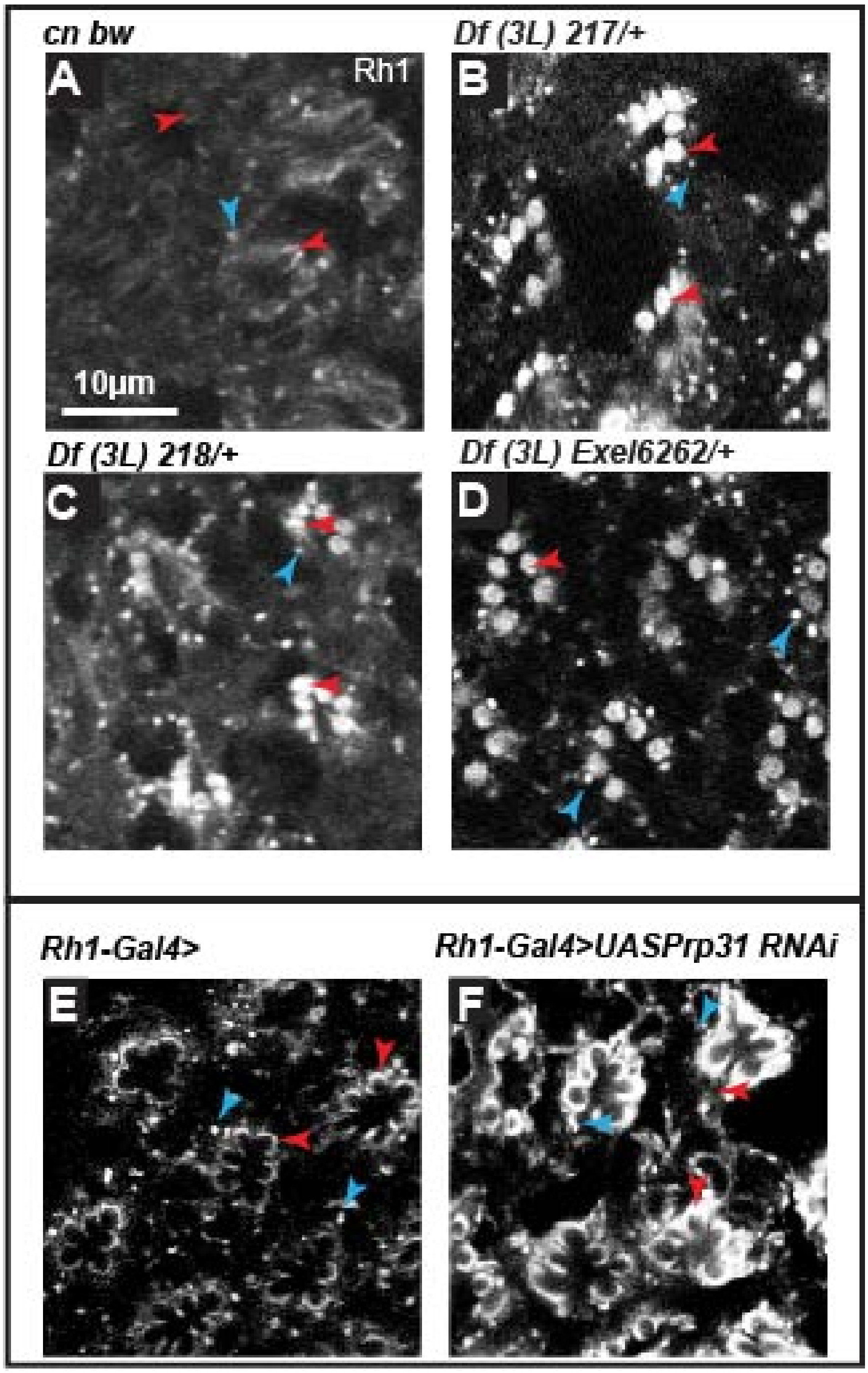

Figure S4

Figure S4: Increased Rh1 immunostaining in eyes of *Prp31* hemizygous animals and upon RNAi knockdown of *Prp31*

Representative confocal images of 1µm optical sections from 12µm cross-sections of eyes of adult males with the genotypes indicated, stained with anti-Rh1. Red arrowheads indicate Rh1 staining in the rhabdomere and blue arrowheads indicate intracellular Rh1. Rh1 staining is more intense in the rhabdomeric membrane of *Prp31* hemizygous animals (B-D) as compared to the genetic control *cn bw* (A). Increased Rh1 immunostaining intensity is also observed upon knocking-down *Prp31* by RNAi (E) as compared to its genetic control (F). All images were taken using the same settings for respective control and experimental animals. Scale bar = 10 µm. Complete genotypes can be found in Suppl. Table S1.

Biology Open • Supplementary information

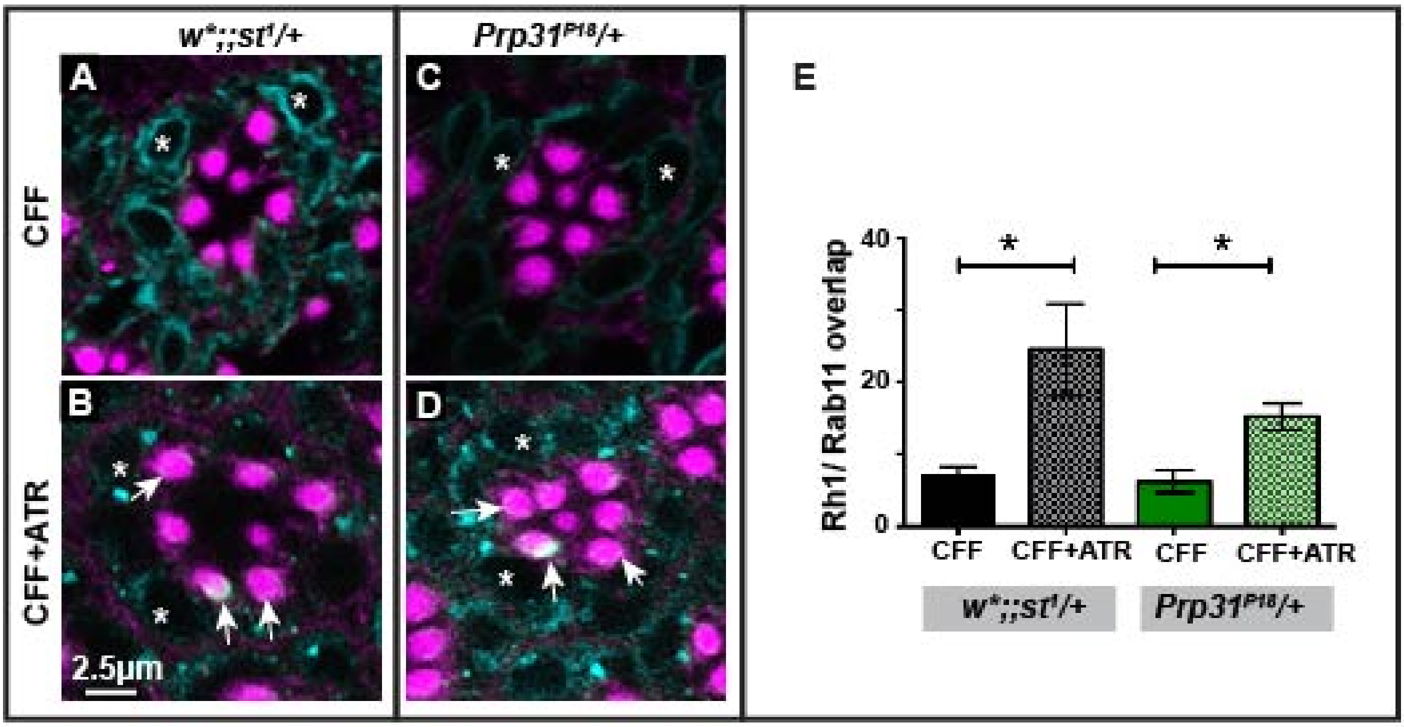

Figure S5

Figure S5: No significant changes in Rh1 trafficking to rhabdomere via Rab11

A-D are confocal images of 12µm cross sections of controls (*w*;;st*^{1}/+, A, B) and mutant (*Prp31*^{P18}/+, C, D) raised on carotenoid free food (CFF, A, C) and 180 minutes after supplementation with All trans retinal (ATR) and blue light exposure (CFF+ATR, B, D). Sections are double labelled with phalloidin (magenta) and an antibody against Rh1 (cyan). With CFF (A, C), Opsin is stuck in the perinuclear endoplasmic reticulum surrounding the nuclei (*). Upon release of Rh1 from the ER (CFF+ATR), it is visible in the inner rim of the rhabdomeres, both in the control and in the mutant (arrows, B, D).

E: Graph quantifying the extent of overlap between Rh1 and Rab11. Bars are mean ± SEM for the percent overlap of Rh1/Rab11 compartments (post-Golgi trafficking of Rh1). With the release of Rh1 from ER following blue light induced chromophore supply (CFF+ATR), a significant increase (*) in the overlap between Rh1 and Rab11 in controls and in mutant eyes is observed, both in the control and in the mutant.

Biology Open • Supplementary information

Table S1: List of Genotypes

Figure	Genotype	Identifier
**Figure 1**	*w*;;*	*w**
	$w^*;;st^1/+$	$w^*;;st^1/+$
	$w^*;;Prp31^{P18}, st^1/+$	$Prp31^{P18}/+$
	$w^*;;Prp31^{P17}, st^1/+$	$Prp31^{P17}/+$
**Figure 2**	*Oregon R (+/+;+;+)*	+
	w;;*	*w**
	$w^*;;st^1/+$	$w^*;;st^1/+$
	$w^*;;Prp31^{P18}, st^1/+$	$Prp31^{P18}/+$
	$w^*;;Prp31^{P17}, st^1/+$	$Prp31^{P17}/+$
	$w^*;;crb^{11A22}$ mosaics (females)	crb^{11A22}
	$w^*;;crb^{p13A9}$ (females)	crb^{p13A9}
**Figure 3**	$GMR\text{-}w^{IR}$*; Rh1-Gal4/+; UAS Dicer-2/+*	*Rh1-Gal4>*
	$GMR\text{-}w^{IR}$*; Rh1-Gal4/+; UAS Dicer-2/UAS Prp31 RNAi* *(VDRC LineID: 35131)*	*Rh1-Gal4> UASPrp31 RNAi*
**Figure 4**	*;cn, bw;*	*cn bw*
	;cn, bw;Df (3L) Exel 6262/+	*Df (3L) Exel 6262/+*
	;cn, bw;Df (3L) ED217/+	*Df (3L) ED217/+*
	;cn, bw;Df (3L) ED218/+	*Df (3L) ED218/+*
**Figure 5**	$GMR\text{-}w^{IR}$*;GMR-Gal4/+;+*	*GMR-Gal4>*
	$GMR\text{-}w^{IR};GMR\text{-}Gal4/+;Prp31^{P18}, st^1/+$	$GMR\text{-}Gal4>; Prp31^{P18}/+$
	$GMR\text{-}w^{IR};GMR\text{-}Gal4, UAS\ st/+;Prp31^{P18}, st^1/+$	$GMR\text{-}Gal4>UAS\ st; Prp31P^{18}/+$
**Figure 6**	*w*;;*	*w**
	$w^*;;st^1/+$	$w^*;;st^1/+$
	$w^*;;Prp31^{P18}, st^1/+$	$Prp31^{P18}/+$
	$w^*;;st^1/ st^1$	$w^*;;st^1/ st^1$
	$w^*;;Prp31^{P18}, st^1/ Prp31^{P18}, st^1$	$Prp31^{P18}/Prp31^{P18}$
**Figure 7**	*w*;;*	*w**
	$w^*;;Prp31^{P18}, st^1/+$	$Prp31^{P18}/+$
	$w^*;;crb^{11A22}$ mosaics (females)	crb^{11A22}
**Figure S2**	$w^*;;Prp31^{P18}, st^1/ Prp31^{P18}, st^1$	$Prp31^{P18}/Prp31^{P18}$
	;cn, bw;	*cn bw*
	;cn, bw;Df (3L) Exel 6262/+	*Df (3L) Exel 6262/+*
	;cn, bw;Df (3L) ED217/+	*Df (3L) ED217/+*

Biology Open • Supplementary information

	;cn, bw/cn, bw;Df (3L) ED217/ Prp31^{P18}, st^{1}	*Df (3L) ED217/ Prp31^{P18}*
	;cn, bw/cn, bw;Df (3L) ED217/ Prp31^{P18}, st^{1}	*Df (3L) ED217/ Prp31^{P18}*
	;cn, bw/cn, bw;Df (3L) ED217/ Prp31^{P17}, st^{1}	*Df (3L) ED217/ Prp31^{P17}*
**Figure S3**	*w*;;*	*w**
	w;;Prp31^{P18}, st^{1}/+*	*Prp31^{P18}/+*
**Figure S4**	*w*;;*	*w**
	;cn, bw;Df (3L) Exel 6262/+	*Df (3L) Exel 6262/+*
	;cn, bw;Df (3L) ED217/+	*Df (3L) ED217/+*
	GMR-w^{IR}; Rh1-Gal4/+; UAS Dicer-2/+	*Rh1-Gal4>*
	GMR-w^{IR}; Rh1-Gal4/+; UAS Dicer-2/UAS Prp31 RNAi (VDRC LineID: 35131)	*Rh1-Gal4> UASPrp31 RNAi*
**Figure S5**	*w*;;st^{1}/+*	*w*;;st^{1}/+*
	w;;Prp31^{P18}, st^{1}/+*	*Prp31^{P18}/+*

(all samples were age-matched males unless specified otherwise)

Biology Open • Supplementary information

Table S2: Outcome of statistical significance tests for quantitative data when comparing (the frequency of ommatidia with 7 rhabdomeres) between genotypes.

Solid black line indicates the pair-wise comparison

* next to the black lines: $p<0.05$ and "n.s." indicates no significant differences

Figure 2

(ANOVA followed by Post hoc Tukey Test)

+	] n.s.					
*w**		] *				
w;;st*^{1}/+			]*			
Prp31^{P18}/+				]*		
Prp31^{P17}/+					]*	] *
crb^{11A22}						
crb^{p13A9}						

Figure 3

(Unpaired Student's t Test)

Rh1-Gal4>	]*
Rh1-Gal4> UASPrp31 RNAi	

Figure 4

(ANOVA followed by Post hoc Tukey Test)

cnbw	] *		
Df (3L) Exel 6262/+		] n.s.	
Df (3L) ED217/+			] *
Df (3L) ED218/+			

Figure 5

(ANOVA followed by Post hoc Tukey Test)

GMR-Gal4>	] *	
GMR-Gal4>& Prp31^{P18}/+		] *
GMR-Gal4>UAS st & Prp31^{P18}/+		

Biology Open • Supplementary information

**Figure 7** (ANOVA followed by Post hoc Tukey Test)		
*w**	n.s. (*w** vs *Prp31*^{P18}/+)	n.s. (*w** vs *crb*^{11A22})
Prp31^{P18}/+		
crb^{11A22}		

Biology Open • Supplementary information

Table S3: Details of Primers used in Real Time qRT-PCR experiments

***ninaE/opsin 1* mRNA**	**Targeted amplicon**	**Primer pairs (sequence 5'-3')**
	Within exon 1	GTTTCCAACGACCAATCGCC<br>CTGCGTCACTATATCCCGCC
	Within exon 2	GACATATACGCCGGACTGGG<br>TTGGTAGCGATCCAGGGAGA
	Within exon 3	GGAGGGTAACCTGACCTCGT<br>GATCAGGTATGAGCGTGGGT
	Within exon 4	TGAATGTCAAGTCCCTCCGC<br>GACCAGGTATGGTGTCCACG
	Within exon 5	AATATCGCCTGGCCCTCAAG<br>GCATCGCTCGATTTGCCATC
	Junction of exon 1-exon 2	TAGTGACGCAGCCAGTAACC<br>TGTCCACCACCGATCCATTG
	Junction of exon 2-exon 3	TTCGGCTGGAGCAGGTATG<br>GTATGAGCGTGGGTTCCAGT
	Junction of exon 3-exon 4	TGGTTCATCATTGCTGCTGTC<br>TGACATTCATCTTCTTGGCCTG
	Junction of exon 4-exon 5	TATACGGCATCAGCCATCCG<br>ATCGTCGACCTTGCCAAAGA
**Rh1 trafficking regulators**	**Target Gene**	**Primer pairs (sequence 5'-3')**
	CG3911/Bet3	ATGTCACGACAAGCCTCTCG<br>GAGTGCTCCGTAGGTGAGT
	twf	CCCTTGGCGTGGAGGTTGTTA<br>AAGAAGGCTTCGGTCAGCT
	CdGAPr	TACAGCCGCTGAATAGCAAATC<br>TGGACCCTTTCGTAGTGGAAG
**Reference Gene**	**Target Gene**	**Primer pairs (sequence 5'-3')**
	Gapdh1	TAAATTCGACTCGACTCACGGT<br>CTCCACCACATACTCGGCTC
